# Oral anticoagulants for prevention of stroke in atrial fibrillation: systematic review, network meta-analysis, and cost effectiveness analysis

**DOI:** 10.1136/bmj.j5058

**Published:** 2017-11-28

**Authors:** José A López-López, Jonathan A C Sterne, Howard H Z Thom, Julian P T Higgins, Aroon D Hingorani, George N Okoli, Philippa A Davies, Pritesh N Bodalia, Peter A Bryden, Nicky J Welton, William Hollingworth, Deborah M Caldwell, Jelena Savović, Sofia Dias, Chris Salisbury, Diane Eaton, Annya Stephens-Boal, Reecha Sofat

**Affiliations:** 1 Department of Population Health Sciences, Bristol Medical School, University of Bristol, Oakfield House, Oakfield Grove, Bristol BS8 2BN, UK; 2 National Institute for Health Research Bristol Biomedical Research Centre, Oakfield House, Oakfield Grove, Bristol BS8 2BN, UK; 3 Faculty of Population Health Sciences, University College London, London, UK; 4 The National Institute for Health Research Collaboration for Leadership in Applied Health Research and Care West (NIHR CLAHRC West) at University Hospitals Bristol NHS Foundation Trust, Bristol, UK; 5 University College London Hospitals NHS Foundation Trust, London, UK; 6 Royal National Orthopaedic Hospital NHS Trust, London, UK; 7 AntiCoagulation Europe, Bromley, Kent, UK; 8 Thrombosis UK, Llanwrda, UK

## Abstract

**Objective:**

To compare the efficacy, safety, and cost effectiveness of direct acting oral anticoagulants (DOACs) for patients with atrial fibrillation.

**Design:**

Systematic review, network meta-analysis, and cost effectiveness analysis.

**Data sources:**

Medline, PreMedline, Embase, and The Cochrane Library.

**Eligibility criteria for selecting studies:**

Published randomised trials evaluating the use of a DOAC, vitamin K antagonist, or antiplatelet drug for prevention of stroke in patients with atrial fibrillation.

**Results:**

23 randomised trials involving 94 656 patients were analysed: 13 compared a DOAC with warfarin dosed to achieve a target INR of 2.0-3.0. Apixaban 5 mg twice daily (odds ratio 0.79, 95% confidence interval 0.66 to 0.94), dabigatran 150 mg twice daily (0.65, 0.52 to 0.81), edoxaban 60 mg once daily (0.86, 0.74 to 1.01), and rivaroxaban 20 mg once daily (0.88, 0.74 to 1.03) reduced the risk of stroke or systemic embolism compared with warfarin. The risk of stroke or systemic embolism was higher with edoxaban 60 mg once daily (1.33, 1.02 to 1.75) and rivaroxaban 20 mg once daily (1.35, 1.03 to 1.78) than with dabigatran 150 mg twice daily. The risk of all-cause mortality was lower with all DOACs than with warfarin. Apixaban 5 mg twice daily (0.71, 0.61 to 0.81), dabigatran 110 mg twice daily (0.80, 0.69 to 0.93), edoxaban 30 mg once daily (0.46, 0.40 to 0.54), and edoxaban 60 mg once daily (0.78, 0.69 to 0.90) reduced the risk of major bleeding compared with warfarin. The risk of major bleeding was higher with dabigatran 150 mg twice daily than apixaban 5 mg twice daily (1.33, 1.09 to 1.62), rivaroxaban 20 mg twice daily than apixaban 5 mg twice daily (1.45, 1.19 to 1.78), and rivaroxaban 20 mg twice daily than edoxaban 60 mg once daily (1.31, 1.07 to 1.59). The risk of intracranial bleeding was substantially lower for most DOACs compared with warfarin, whereas the risk of gastrointestinal bleeding was higher with some DOACs than warfarin. Apixaban 5 mg twice daily was ranked the highest for most outcomes, and was cost effective compared with warfarin.

**Conclusions:**

The network meta-analysis informs the choice of DOACs for prevention of stroke in patients with atrial fibrillation. Several DOACs are of net benefit compared with warfarin. A trial directly comparing DOACs would overcome the need for indirect comparisons to be made through network meta-analysis.

**Systematic review registration:**

PROSPERO CRD 42013005324.

## Introduction

The prevalence of atrial fibrillation roughly doubles with each decade of age, rising to almost 9% at 80-90 years.^1^ ^2^ ^3^ Atrial fibrillation increases the risk of thromboembolic stroke fivefold, as a result of blood pooling in the left atrium and systemic embolisation to the brain. More than a fifth of the 130 000 annual strokes in England and Wales are attributed to atrial fibrillation (annual incidence of 114 in 100 000).^4^ Patients with thromboembolic stroke from atrial fibrillation have higher mortality, higher morbidity, and longer hospital stays than patients with other stroke subtypes.^1^

The oral anticoagulant warfarin, a vitamin K antagonist, is effective for prevention of stroke in patients with atrial fibrillation.^5^ However, bleeding associated with warfarin is among the top five reasons for hospital stays, secondary to adverse drug effects, in England.^6^ Warfarin has a narrow therapeutic index as well as problematic drug and dietary interactions. The international normalised ratio (INR) requires monitoring (through hospital, primary care, anticoagulation clinics based in pharmacies, or by home monitoring with clinic support) to ensure optimal warfarin efficacy while limiting the risk of bleeding. Such monitoring is a large proportion of the overall cost of warfarin use, estimated at £90 million annually in the National Health Service (NHS) in England, Wales, and Northern Ireland.^7^ Because of its perceived risk and inconvenience warfarin is underused, particularly in those at high risk of stroke.^8^ It is estimated that only 46% of those who should be on warfarin are receiving it, with up to 40% of these not in the optimal therapeutic range of 2.0-3.0 INR units.^7^

Direct acting (non-vitamin K antagonist) oral anticoagulants (DOACs) overcome some of the limitations of warfarin, offering important benefits that can improve quality of life for patients and their carers. The class includes factor II inhibitors (eg, dabigatran) and factor Xa inhibitors (eg, apixaban, betrixaban, edoxaban, and rivaroxaban). DOACs do not require monitoring, have a more predictable pharmacokinetic (dosing) profile, and have fewer interactions with other drugs. Furthermore, they have rapid onset and offset of action, avoiding loading and use of low molecular weight heparin (LMWH) for bridging. However, their cost is substantially higher than that of warfarin and will remain so until market exclusivity periods end and generic products become available (indicative dates 2022, 2018, 2023, and 2020 for apixaban, dabigatran, edoxaban, and rivaroxaban respectively). Potential limitations of DOACs include class specific or drug specific cautions and contraindications, potential for subtherapeutic dosing, reduced adherence owing to lack of regular monitoring, absence of (or limited experience with) drug products to reverse the anticoagulant effects, the cost of maintaining stocks of different anticoagulants, and the potential for prescribing errors owing to unfamiliarity.^9^

Systematic reviews of randomised trials of DOACs have concluded that they have a similar efficacy to warfarin but may have some advantages with respect to the risk of bleeding.^10^ ^11^ ^12^ The DOACs have also been evaluated individually by the National Institute for Health and Care Excellence (NICE), and the respective technology appraisals have recommended their use.^7^ However, no trials have directly compared different DOACs with each other, so it is difficult to determine which drug should be recommended as a first choice for most patients. It also remains unclear whether the higher costs of DOACs are offset by improved efficacy benefits or a reduced need for therapeutic monitoring, or both. In addition, the effects of DOACs may have been overestimated in clinical trials because some patients randomised to warfarin were not maintained within the therapeutic INR target of 2.0-3.0.^13^ ^14^ ^15^ We conducted a systematic review, network meta-analysis, and cost effectiveness analysis to compare DOACs with each other and with warfarin for prevention of stroke in patients with atrial fibrillation, and recommend a rank order based on efficacy, safety, and cost.

## Methods

### Study eligibility and selection

Our systematic review was prospectively registered with the National Institute for Health Research prospective register. Methods were in accordance with guidelines of the University of York Centre for Reviews and Dissemination^16^ and Cochrane.^17^ A detailed report of the methods and results is available elsewhere.^18^

We included phase II or phase III randomised controlled trials using either a superiority or non-inferiority design, that evaluated the use of a direct acting oral anticoagulant (DOAC), vitamin K antagonist, or antiplatelet agent for prevention of stroke in patients with atrial fibrillation. We included adults with non-valvular atrial fibrillation eligible for oral anticoagulation. Trials in participants only eligible for parenteral anticoagulation were excluded. Unless otherwise specified, anticoagulation services may have been delivered in hospital, primary care, or pharmacy based clinics or through home monitoring and telephone support. The review was not limited to NHS anticoagulation services. 

We focused on five DOACs; four direct factor Xa inhibitors: apixaban, betrixaban, edoxaban, and rivaroxaban, and one direct factor II (thrombin) inhibitor: dabigatran. The following direct factor Xa inhibitors were excluded: eribaxaban because the current stage of development was unclear; otamixaban because it is administered parenterally; darexaban (YM150) because it has been discontinued; and LY517717 and letaxaban (TAK442) because no information on any further clinical development was available. Two factor II inhibitors were also excluded: ximelagatran because it has been withdrawn as a result of liver toxicity and AZD0837 because it has been discontinued. Furthermore, we excluded trials comparing only different doses of the same drug, trials reporting only short term follow-up data (less than three months), trials of warfarin with target INR of 2.0 or less, and one trial that included only patients who were without thrombogenic characteristics as detected using transoesophageal echocardiography.

To determine the comparator interventions, we constructed network plots to ensure they would provide information on the relative effectiveness of the DOACs of interest. Comparators were therapeutic doses of warfarin or other vitamin K antagonist (with optimal INR range 2.0-4.0), as well as aspirin and clopidogrel. We excluded studies evaluating a fixed dose of warfarin, and where warfarin administration for all patients had suboptimal target INR compared with UK guidelines (INR 2.0–3.0).

The main outcomes of our interest were decided from the network meta-analyses and chosen based on three considerations:^1^ their clinical importance;^2^ the consistency of reporting across studies included in the network; and the amount of data available for inclusion in network meta-analysis.^3^ Outcomes extracted included all stroke, stroke or systemic embolism, ischaemic stroke, haemorrhagic stroke, myocardial infarction, all-cause mortality, all bleeding, minor bleeding, major bleeding, intracranial bleeding, gastrointestinal bleeding, and clinically relevant bleeding. Where necessary we derived numbers of compound events from components reported in trial publications.

We screened the studies included in previously published network meta-analysis of DOACs against our eligibility criteria. We developed searches to identify additional studies published from 2010 onwards, implemented in Medline (see web appendix 1), PreMedline, Embase, and The Cochrane Library. We also searched the NHS Economic Evaluation Database and NICE Technology Appraisals, within The Cochrane Library. We applied no restrictions on language. We sought information on studies in progress, unpublished research, or research reported in the grey literature and searched ClinicalTrials.gov (to August 2016). We screened reference lists of retrieved studies and relevant review articles.

### Collection of data and assessment of the risk of bias

Two members of the review team independently screened titles and abstracts. We assessed full texts of all potentially relevant reports for inclusion, having collated multiple reports from the same studies. We extracted the following data: study details (identifier, study design, location, year, length of follow-up, and industry sponsorship); participant details (number of participants, age, and sex); intervention details (drug name, dose, and timing); comparator details; details relevant to the risk of bias assessment (including adherence to and withdrawal from randomised allocation); and effect modifiers. Multiple reports from a study informed a single data extraction form. We extracted dichotomous data as number of events in intervention and control groups and numbers of participants, and we sought details of follow-up time. We also extracted estimates of hazard ratios and their confidence intervals where available. We extracted intention to treat data where these were reported. Otherwise we extracted the data as reported (often a modified intention to treat based on, eg, all patients who received at least one dose of the study drug).

Data extraction and risk of bias assessments using the Cochrane tool were carried out by one reviewer (GNO) in a Microsoft Access data collection form, and checked by a second reviewer (PB).^19^ Disagreements were resolved by consensus or by referral to a third reviewer (PAD or JS) where necessary.

### Data synthesis and statistical analysis

We generated network plots of comparisons to illustrate which interventions had been compared within randomised trials (direct comparisons). Different doses or frequencies of administration (once daily or twice daily) of DOACs were analysed separately and hence appear as separate points in network plots. We defined two independent nodes for warfarin interventions, labelled as warfarin (INR 2.0-3.0) and warfarin (INR 3.0-4.0) respectively. The first of these formed the reference treatment across all networks. We also included in warfarin (INR 2.0-3.0) some interventions with an INR range of 2.5-3.5 or 2.0-4.5. In some trials the INR range for some patients in the warfarin arm was subtherapeutic (below 2.0), so that the total INR range was 1.6-3.0. These interventions were excluded from the main analysis, but merged with the INR 2.0-3.0 node in a sensitivity analysis.

We considered two separate nodes for antiplatelets, less than 150 mg once daily and 150 mg or more once daily. The dose range considered in the AVERROES^20^ trial (81-324 mg once daily) was much wider than in any other trial, and we included this intervention in the lower dose node (<150 mg once daily) because some patients from that study had received a low daily dose. As a sensitivity analysis, we excluded the AVERROES trial from the network.

Where outcome data were presented for multiple time points, we took the longest period of follow-up. For stroke or systemic embolism, we used the total number of stroke events if the former was not reported. When clinically relevant bleeding was not reported, we calculated it as the sum of the major bleeding and clinically relevant non-major bleeding events.

In the primary network meta-analyses, we treated data as binomial, modelling the number of events out of the total number of participants using a logistic model. We omitted trials with no events in any arm and where there were events in at least one arm of a trial but no events in one or more other arms, we added 0.5 events to all cells in the 2×2 table for that trial. As sensitivity analyses, we also undertook separate analyses for all outcomes where we took into account the different follow-up periods and the different reporting patterns considered across studies (see web appendix 3 and 4).

We conducted both standard meta-analyses of each pairwise direct comparison between interventions, and a network meta-analysis combining results of all these comparisons in one analysis, exploiting both the direct comparisons within trials and the indirect comparisons across trials for each outcome. The network meta-analyses used a logistic regression approach, implemented in a Bayesian framework using WinBUGS software (version 1.4.3).^21^ We used a fixed effect model, because the number of studies for each comparison was small. We present results as odds ratios with 95% confidence intervals and as rankograms displaying the probability that each intervention evaluated in phase III trials is ranked highest, second highest, and so on, for preventing each outcome. To assess consistency among sources of evidence, we back calculated the indirect comparisons of interventions from the network meta-analysis results and the direct comparisons. We first tabulated intervention effects for each DOAC against warfarin (mostly based on direct comparisons), and then comparisons, derived from the network meta-analysis, between the DOAC dosing strategies that had been evaluated in a phase III trial. 

We prespecified important characteristics to be age, sex, ethnicity or race, body mass index or weight, renal status or creatinine clearance, blood pressure, diabetes mellitus, hypertension, previous thrombotic event, liver disease, chronic heart failure, cancer, pregnancy, intervention dose, mean time in warfarin therapeutic range, CHADS_2_ score, CHA_2_DS_2_-VASc score, HAS-BLED score, history of previous stroke or transient ischaemic attack, previous myocardial infarction, and summary assessment of the risk of bias for each outcome. We used meta-regression to determine the influence of these potential effect modifiers.

### Cost effectiveness analysis

We evaluated the most cost effective first-line (initially used) anticoagulant for the prevention of ischaemic stroke in patients with atrial fibrillation, from the perspective of the UK National Health Service (NHS). Only recommended doses were considered. Betrixaban was excluded from this analysis because of insufficiently precise evidence regarding efficacy. The base case was a cohort aged 70, modelled to the end of life. We used a discrete time Markov multistate model, with a cycle length of three months.^22^ The main assumptions and structure of the model are provided in web appendix 2. We estimated expected lifetime total costs and quality adjusted life years (QALYs) for a patient with atrial fibrillation, aged 70, beginning each first-line anticoagulation strategy. We estimated the net monetary benefit for each strategy using the willingness to pay threshold of £20 000 (the amount the UK NHS is willing to pay for one year of perfect health, which is one QALY). Expected incremental costs, QALYs, and net benefit for each preferred strategy compared with warfarin INR 2.0-3.0 were also estimated. We conducted a wide range of sensitivity analyses, described in web appendix 2. A key sensitivity analysis explored the price at which DOACs would have to be sold to become most cost effective.

### Patient involvement

Two representatives (DE and ASB) of patient group charities (AntiCoagulation Europe and Thrombosis UK) participated in the design (as co-applicants on the grant application), conduct (including attending project meetings), reporting, and interpretation of the results of this study, and are included as co-authors of this paper. It was not evaluated whether the studies included in the review had any patient involvement****

## Results

### Studies included

**Fig 1:**
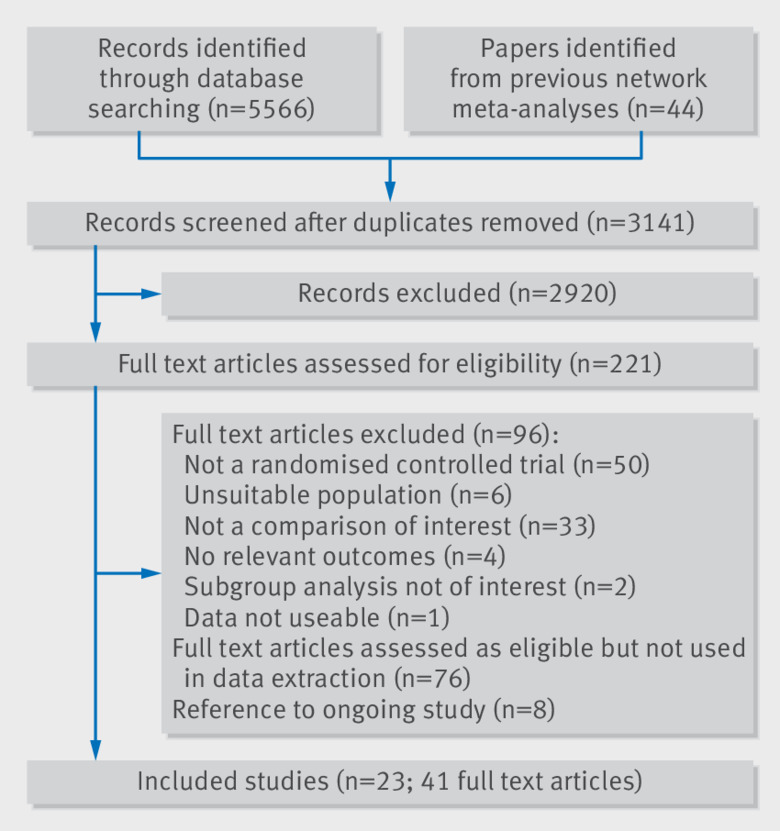
PRISMA flowchart for review of prevention of stroke in patients with atrial fibrillation

Figure 1 shows how we identified 23 completed, eligible, randomised trials involving 94 656 patients (see web appendix 3 for a summary of trial characteristics). All reports were written in English except for one paper written in Chinese that was translated with assistance from a native Chinese speaker.^23^ Sixteen trials, involving 97% of patients, were phase III. Where reported, the mean age of included patients ranged from 63.3 to 81.5 years (median 70.0 years); proportions of male patients from 44.9% to 82.9% (63.3%); and mean body mass index (BMI) from 24.4 to 30.5 kg/m^2^ (28.0 kg/m^2^). The percentage of patients with previous stroke (5.0% to 63.8%, median 20.2%), hypertension (38.0% to 93.7%, 73.8%), and chronic heart failure (0% to 100%, 32%) varied across the studies. Mean time in therapeutic range for warfarin arms ranged from 45.1% to 83.0% (median 63.8%) of the duration of treatment.

Thirteen studies (six phase III 15 24 25 26 27 28 and seven phase II 13 14 29 30 31 32 33) examined one of the following direct acting oral anticoagulants (DOACs): apixaban,^24^ ^25^ ^31^ betrixaban,^32^ dabigatran,^13^ ^28^ ^33^ edoxaban,^14^ ^26^ ^29^ ^30^ and rivaroxaban.^15^ ^27^ Treatment durations ranged from three to 30 months: outcomes were reported at the end of the treatment period. Pharmaceutical companies sponsored 15 studies, including all those examining DOACs. Sponsor details were not reported in two studies. Table 1 shows the number of patients analysed and the number of events for the main outcomes reported in each trial (event counts for other outcomes are in web appendix 4).

**Table 1:** Number of events for each main outcome reported by 23 randomised trials in prevention of stroke in patients with atrial fibrillation

Study	Study size*	Reporting pattern†		Stroke					Myocardial infarction	All-cause mortality		Bleeding					
				All	or SE	Ischaemic	Haemorrhagic					All	Minor	Major	IC	GI	CR
ACTIVE W	6706	1		159	NA	132	20		59	317		1199	1049	194	NA	NA	NA
AF-ASA-VKA-CHINA	101	2		NA	18	14	NA		5	4		14	9	3	NA	1	
AF-DABIG-VKA-JAPAN	166	2		NA	1	NA	NA		NA	NA		45	NA	3	NA	NA	14
AF-EDOX-VKA-ASIA	234	2		NA	0	NA	NA		NA	NA		57	48	2	NA	1	11
AF-EDOX-VKA-JAPAN	519	2		NA	1	NA	NA		NA	NA		115	NA	5	NA	NA	20
AF-EDOX-VKA-MULTI	1143	2		NA	11	NA	NA		5	NA		114	52	13	NA	NA	62
AF-VKA-ASA-CHINA	440	2		10	NA	9	1		NA	11		NA	25	8	NA	7	NA
AFASAK	671	2		20	NA	NA	NA		NA	15		23	NA	NA	NA	5	NA
AFASAK II	339	1		19	22	8	2		8	31		NA	68	9	3	NA	NA
ARISTOTLE	18140	3		449	477	337	118		192	1272		5416	NA	789	174	224	1490
ARISTOTLE-J	218	2		NA	3	1	NA		0	0		41	36	1	NA	1	6
AVERROES	5599	1		154	164	128	15		52	251		NA	341	83	24	26	263
BAFTA	973	1		94	NA	NA	NA		30	215		NA	NA	50	NA	NA	NA
Chinese ATAFS	704	2		23	NA	NA	NA		NA	12		NA	NA	NA	NA	NA	NA
ENGAGE AF-TIMI 48	21 026	2		958	1016	804	169		443	2349		NA	1851	1196	234	551	4450
EXPLORE-Xa	508	2		2	NA	2	NA		0	2		118	109	8	NA	NA	18
J-ROCKET AF	1278	2		31	33	24	7		4	12		NA	NA	NA	15	18	262
PATAF	272	1		7	NA	7	NA		5	29		NA	NA	NA	NA	11	NA
PETRO	515	2		NA	2	NA	NA		NA	NA		88	NA	4	NA	NA	36
RE-LY	18 113	2		NA	519	389	71		270	1371		NA	5284	1162	150	435	NA
ROCKET AF	14 236	2		405	575	310	NA		227	458		NA	NA	781	139	378	2924
SPAF II	1100	3		NA	67	63	NA		34	127		NA	NA	NA	18	NA	NA
WASPO	75	2		0	NA	NA	NA		NA	3		NA	10	3	NA	3	NA
Total	93 076			2331	2909	2228	403		1334	6479		7230	8882	4314	757	1661	9556

SE=systemic embolism; IC=intracranial; GI=gastrointestinal; CR=clinically relevant; NA=not available * Study sizes are based on the arms that were relevant to the purpose of this review.  †1=Number of patients whose first event is of a given type, patients censored thereafter; 2=Number of patients experiencing at least one event of each given type; 3=Total number of events of each type.

### Risk of bias in included studies

The risk of bias judgments for studies contributing to analyses of each outcome are presented in web appendix 5. Most studies were judged to be at a low or unclear risk of bias for sequence generation and at low risk of bias for allocation concealment. Most studies were open label and were judged to be at high risk of bias for blinding of participants and staff. Most studies were judged to be at low risk of bias for blinding of outcome assessment and for incomplete outcome data.

### Efficacy and safety results

**Fig 2:**
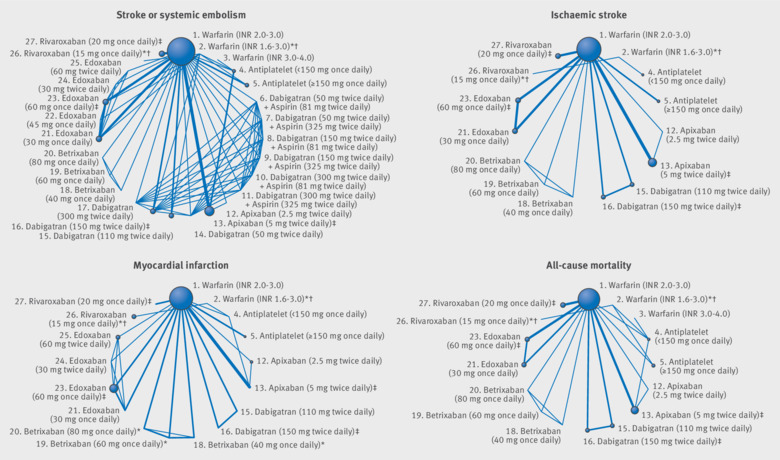
Network plots of stroke or systemic embolism, ischaemic stroke, myocardial infarction, and all-cause mortality outcomes for review of prevention of stroke in patients with atrial fibrillation. Line thickness is proportional to the number of patients that contributed to the comparison *Doses of direct acting oral anticoagulants (DOACs) that were excluded from the primary analysis owing to not being considered to be of interest to inform health decisions in the UK (eg, warfarin interventions using subtherapeutic INR ranges), the total number of events was zero so they are uninformative, or they did not connect with the other trials in the network. †Excluded doses of DOACs that were included in sensitivity analyses. ‡Recommended doses of DOACs evaluated in a phase III trial; these are interventions of primary interest.

**Fig 3:**
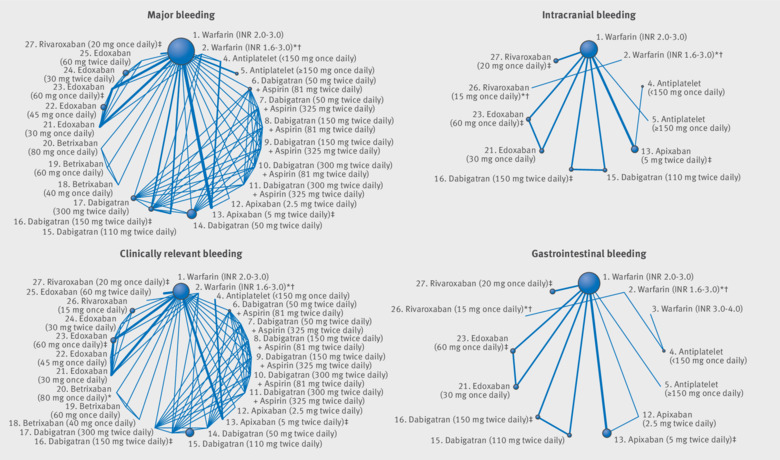
Network plots of bleeding outcomes for review of prevention of stroke in patients with atrial fibrillation. Line thickness is proportional to the number of patients that contributed to the comparison *Doses of direct acting oral anticoagulants (DOACs) that were excluded from the primary analysis owing to not being considered to be of interest to inform health decisions in the UK (eg, warfarin interventions using subtherapeutic INR ranges), the total number of events was zero so they are uninformative, or they did not connect with the other trials in the network. †Excluded doses of DOACs that were included in sensitivity analyses. ‡Recommended doses of DOACs evaluated in a phase III trial; these are interventions of primary interest.

A total of 27 different interventions were included in the network: the direct comparisons made for different outcomes are shown in figure 2 (efficacy outcomes) and figure 3 (safety outcomes). Table 2 shows evidence that apixaban 5 mg twice daily (odds ratio 0.79, 95% confidence interval 0.66 to 0.94), dabigatran 150 mg twice daily (0.65, 0.52 to 0.81), edoxaban 60 mg once daily (0.86, 0.74 to 1.01), and rivaroxaban 20 mg once daily (0.88, 0.74 to 1.03) all reduce the risk of stroke or systemic embolism compared with warfarin INR 2.0-3.0. The risk of ischaemic stroke was lower for dabigatran (0.76, 0.58 to 0.98) but higher for antiplatelet interventions (<150 mg once daily: 1.61, 1.25 to 2.07 and ≥150 mg once daily: 1.88, 1.40 to 2.51) than for warfarin INR 2.0-3.0. Comparing DOACs, there was evidence of a higher risk of stroke or systemic embolism with edoxaban 60 mg once daily (1.33, 1.02 to 1.75) and rivaroxaban 20 mg once daily (1.35, 1.03 to 1.78) than with dabigatran 150 mg twice daily. There was little evidence that the risk of ischaemic stroke differed between licensed strengths of DOACs.

**Table 2:** Results of the stroke, myocardial infarction, and mortality outcomes for review of prevention of stroke in patients with atrial fibrillation

	Stroke or systemic embolism			Ischaemic stroke			Myocardial infarction			All-cause mortality	
	Type*	Odds ratio (95% CI)		Type	Odds ratio (95% CI)		Type	Odds ratio (95% CI)		Type	Odds ratio (95% CI)
**Comparison with warfarin INR 2.0-3.0 **											
Antiplatelet:											
<150 mg once daily	D and I	1.88 (1.40 to 2.51)		I	2.52 (1.62 to 3.99)		D and I	1.02 (0.64 to 1.64)		D and I	1.08 (0.88 to 1.33)
≥150 mg once daily	D	1.61 (1.25 to 2.07)		D	2.00 (1.51 to 2.67)		D	1.38 (0.94 to 2.03)		D	1.04 (0.87 to 1.25)
Apixaban:											
5 mg twice daily	D	0.79 (0.66 to 0.94)		D	0.92 (0.74 to 1.14)		D	0.87 (0.66 to 1.15)		D	0.88 (0.79 to 0.98)
Dabigatran:											
110 mg twice daily	D	0.90 (0.74 to 1.10)		D	1.14 (0.90 to 1.44)		D	1.32 (0.97 to 1.79)		D	0.91 (0.80 to 1.04)
150 mg twice daily	D	0.65 (0.52 to 0.81)		D	0.76 (0.58 to 0.98)		D	1.29 (0.96 to 1.75)		D	0.88 (0.77 to 1.01)
Edoxaban:											
30 mg once daily	D	1.13 (0.97 to 1.32)		D	1.44 (1.21 to 1.71)		D	1.22 (0.97 to 1.53)		D	0.86 (0.78 to 0.96)
60 mg once daily	D	0.86 (0.74 to 1.01)		D	1.01 (0.84 to 1.21)		D	0.96 (0.75 to 1.22)		D	0.91 (0.82 to 1.01)
Rivaroxaban:											
20 mg once daily	D	0.88 (0.74 to 1.03)		D	0.93 (0.74 to 1.16)		D	0.80 (0.61 to 1.04)		D	0.83 (0.69 to 1.00)
**Comparison between recommended doses of DOACs evaluated in a phase III trial**											
Dabigatran 150 mg twice daily and apixaban 5 mg twice daily	I	0.82 (0.62 to 1.08)		I	0.83 (0.59 to 1.16)		I	1.48 (0.98 to 2.22)		I	1.00 (0.84 to 1.19)
Edoxaban 60 mg once daily and apixaban 5 mg twice daily	I	1.09 (0.87 to 1.39)		I	1.10 (0.83 to 1.46)		I	1.10 (0.76 to 1.58)		I	1.03 (0.89 to 1.20)
Rivaroxaban 20 mg once daily and apixaban 5 mg twice daily	I	1.11 (0.87 to 1.41)		I	1.01 (0.74 to 1.38)		I	0.92 (0.63 to 1.34)		I	0.94 (0.76 to 1.17)
Edoxaban 60 mg once daily and dabigatran 150 mg twice daily	I	1.33 (1.02 to 1.75)		I	1.33 (0.97 to 1.83)		I	0.74 (0.50 to 1.09)		I	1.03 (0.87 to 1.22)
Rivaroxaban 20 mg once daily and dabigatran 150 mg twice daily	I	1.35 (1.03 to 1.78)		I	1.22 (0.87 to 1.73)		I	0.62 (0.41 to 0.93)		I	0.94 (0.74 to 1.18)
Rivaroxaban 20 mg once daily and edoxaban 60 mg once daily	I	1.01 (0.80 to 1.27)		I	0.92 (0.69 to 1.23)		I	0.84 (0.59 to 1.20)		I	0.91 (0.73 to 1.13)

Comparisons for which the ratio between 95% confidence interval limits exceeded nine were considered imprecisely estimated and were not reported in tables, except where they included a recommended dose of a DOAC. DOACs=direct acting oral anticoagulants ^*^Type of evidence: D=direct; I=indirect; D and I=both direct and indirect

There was weak evidence that the risk of myocardial infarction was higher with dabigatran 110 mg twice daily (odds ratio 1.32, 95% confidence interval 0.97 to 1.79), dabigatran 150 mg twice daily (1.29, 0.96 to 1.75), and edoxaban 30 mg once daily (1.22, 0.97 to 1.53) compared with warfarin INR 2.0-3.0. The risk of myocardial infarction was lower with rivaroxaban 20 mg once daily (0.80, 0.61 to 1.04) compared with warfarin INR 2.0-3.0. Between the DOACs, there was weak evidence that the risk of myocardial infarction was higher with dabigatran 150 mg twice daily compared with apixaban 5 mg twice daily (1.48, 0.98 to 2.2), and lower with rivaroxaban 20 mg once daily compared with dabigatran 150 mg twice daily (0.62, 0.41 to 0.93). The risk of all-cause mortality was lower with all the DOAC interventions compared with warfarin INR 2.0-3.0: odds ratios ranged from 0.83 for rivaroxaban 20 mg once daily (95% confidence interval 0.69 to 1.00) to 0.91 for dabigatran 110 mg twice daily (0.80 to 1.04) and edoxaban 60 mg once daily (0.82 to 1.01). There was little evidence of differences between the effects of licensed DOACs on all-cause mortality.

Table 3 shows evidence that apixaban 5 mg twice daily (odds ratio 0.71, 95% confidence interval 0.61 to 0.81), dabigatran 110 mg twice daily (0.80, 0.69 to 0.93), edoxaban 30 mg once daily (0.46, 0.40 to 0.54), and edoxaban 60 mg once daily (0.78, 0.69 to 0.90) all reduce the risk of major bleeding compared with warfarin INR 2.0-3.0. Between the DOACs, there was evidence that the risk of major bleeding was higher with dabigatran 150 mg twice daily compared with apixaban 5 mg twice daily (1.33, 1.09 to 1.62), with rivaroxaban 20 mg twice daily compared with apixaban 5 mg twice daily (1.45, 1.19 to 1.78), and with rivaroxaban 20 mg twice daily compared with edoxaban 60 mg once daily (1.31, 1.07 to 1.59). There was strong evidence that the risk of intracranial bleeding was lower with apixaban 5 mg twice daily, dabigatran 110 mg twice daily, dabigatran 150 mg twice daily, edoxaban 30 mg once daily, edoxaban 60 mg once daily, and rivaroxaban 20 mg once daily compared with warfarin INR 2.0-3.0. For each of these DOACs and doses, except for rivaroxaban 20 mg once daily, the estimated relative risk reduction for intracranial bleeding was more than 50%. There was strong evidence that the risk of intracranial bleeding was lower with apixaban 5 mg twice daily compared with the other doses of licenced strengths of DOACs. The risk of intracranial bleeding was higher with rivaroxaban 20 mg once daily compared with apixaban 5 mg twice daily, dabigatran 150 mg twice daily, and edoxaban 60 mg once daily.

**Table 3:** Results of bleeding outcomes for prevention of stroke in patients with atrial fibrillation

	Major bleeding			Intracranial bleeding			Gastrointestinal bleeding			Clinically relevant bleeding	
	Type*	Odds ratio (95% CI)		Type	Odds ratio (95% CI)		Type	Odds ratio (95% CI)		Type	Odds ratio (95% CI)
**Comparisons with warfarin INR 2.0-3.0 **											
Antiplatelet:											
<150 mg once daily	D and I	0.75 (0.52 to 1.06)		I	0.50 (0.21 to 1.23)		I	1.03 (0.46 to 2.35)		D and I	0.59 (0.45 to 0.77)
≥150 mg once daily	D	1.07 (0.82 to 1.42)		D	0.39 (0.13 to 0.98)		D	1.60 (0.70 to 3.85)		NA	NA
Apixaban:											
5 mg twice daily	D	0.71 (0.61 to 0.81)		D	0.42 (0.30 to 0.58)		D	0.89 (0.68 to 1.15)		D	0.67 (0.60 to 0.75)
Dabigatran:											
110 mg twice daily	D	0.80 (0.69 to 0.93)		D	0.31 (0.19 to 0.47)		D	1.11 (0.87 to 1.42)		NA	NA
150 mg twice daily	D	0.94 (0.81 to 1.08)		D	0.40 (0.27 to 0.59)		D	1.52 (1.20 to 1.91)		D	1.56 (0.50 to 5.74)
Edoxaban:											
30 mg once daily	D	0.46 (0.40 to 0.54)		D	0.31 (0.21 to 0.43)		D	0.67 (0.53 to 0.84)		D	0.59 (0.54 to 0.64)
45 mg once daily	NA	NA		NA	NA		NA	NA		D	1.09 (0.37, 3.04)
60 mg once daily	D	0.78 (0.69 to 0.90)		D	0.46 (0.33 to 0.62)		D	1.22 (1.01 to 1.49)		D	0.84 (0.77 to 0.90)
30 mg twice daily	NA	NA		NA	NA		NA	NA		D	1.97 (1.04 to 3.67)
60 mg twice daily	NA	NA		NA	NA		NA	NA		D	2.76 (1.46 to 5.17)
Rivaroxaban:											
20 mg once daily	D	1.03 (0.89 to 1.18)		D	0.65 (0.46 to 0.91)		D	1.47 (1.20 to 1.81)		D	1.03 (0.95 to 1.11)
Comparison between recommended doses of DOACs evaluated in a phase III trial											
Dabigatran 150 mg twice daily and apixaban 5 mg twice daily	I	1.33 (1.09 to 1.62)		I	0.96 (0.58 to 1.60)		I	1.71 (1.21 to 2.43)		I	2.32 (0.74 to 8.63)
Edoxaban 60 mg once daily and apixaban 5 mg twice daily	I	1.11 (0.92 to 1.35)		I	1.09 (0.69 to 1.70)		I	1.38 (1.00 to 1.92)		I	1.24 (1.09 to 1.42)
Rivaroxaban 20 mg once daily and apixaban 5 mg twice daily	I	1.45 (1.19 to 1.78)		I	1.55 (0.97 to 2.49)		I	1.66 (1.19 to 2.33)		I	1.53 (1.33 to 1.75)
Edoxaban 60 mg once daily and dabigatran 150 mg twice daily	I	0.84 (0.69 to 1.02)		I	1.13 (0.69 to 1.87)		I	0.81 (0.60 to 1.09)		I	0.54 (0.14 to 1.68)
Rivaroxaban 20 mg once daily and dabigatran 150 mg twice daily	I	1.10 (0.90 to 1.34)		I	1.61 (0.96 to 2.72)		I	0.97 (0.71 to 1.33)		I	0.66 (0.18 to 2.07)
Rivaroxaban 20 mg once daily and edoxaban 60 mg once daily	I	1.31 (1.07 to 1.59)		I	1.43 (0.90 to 2.26)		I	1.21 (0.90 to 1.60)		I	1.23 (1.10 to 1.37)

Comparisons for which the ratio between 95% confidence interval limits exceeded nine were considered imprecisely estimated and were not reported in tables, except where they included a recommended dose of a DOAC. NA=not available; DOACs=direct acting oral anticoagulants ^*^Type of evidence: D=direct; I=indirect; D and I=both direct and indirect

There was evidence that the risk of gastrointestinal bleeding was higher with dabigatran 150 mg twice daily (odds ratio 1.52, 95% confidence interval 1.20 to 1.91), edoxaban 60 mg once daily (1.22, 1.01 to 1.49), and rivaroxaban 20 mg once daily (1.47, 1.20 to 1.81) than for warfarin INR 2.0-3.0. The risk of gastrointestinal bleeding was lower with apixaban 5 mg twice daily than with other doses of DOACS.

The risk of clinically relevant bleeding during antiplatelet therapy <150 mg once daily was lower than with warfarin INR 2.0-3.0 (odds ratio 0.59, 95% confidence interval 0.45 to 0.77). There was evidence that the risk of clinically relevant bleeding was also lower with apixaban 5 mg twice daily (0.67, 0.60 to 0.75), edoxaban 30 mg once daily (0.59, 0.54 to 0.64), and edoxaban 60 mg twice daily (0.84, 0.77 to 0.90) than with warfarin INR 2.0-3.0. However, edoxaban 30 mg twice daily and edoxaban 60 mg twice daily substantially increased the risk of clinically relevant bleeding compared with warfarin INR 2.0-3.0**.** Between the DOACs, there was evidence that the risk of clinically relevant bleeding was higher with edoxaban 60 mg once daily compared with apixaban 5 mg twice daily (1.24, 1.09 to 1.42), rivaroxaban 20 mg once daily compared with apixaban 5 mg twice daily (1.53, 1.33 to 1.75), and rivaroxaban 20 mg once daily compared with edoxaban 60 mg once daily (1.23, 1.1 to 1.37). 

In meta-regression analysis there was no evidence of effect modification owing to mean age, percentage of male patients, mean CHADS_2_ score, or mean time in warfarin therapeutic range for the main outcomes (web appendix 6). There were not enough data to analyse the influence of other effect modifiers. Furthermore, we found similar results after merging the warfarin interventions where the INR range was 1.6-3.0 with the reference node (warfarin INR 2.0-3.0), after excluding the unusually large dose range considered for aspirin in the AVERROES trial, and with more elaborate models taking into account the follow-up periods and reporting patterns across studies.

**Fig 4:**
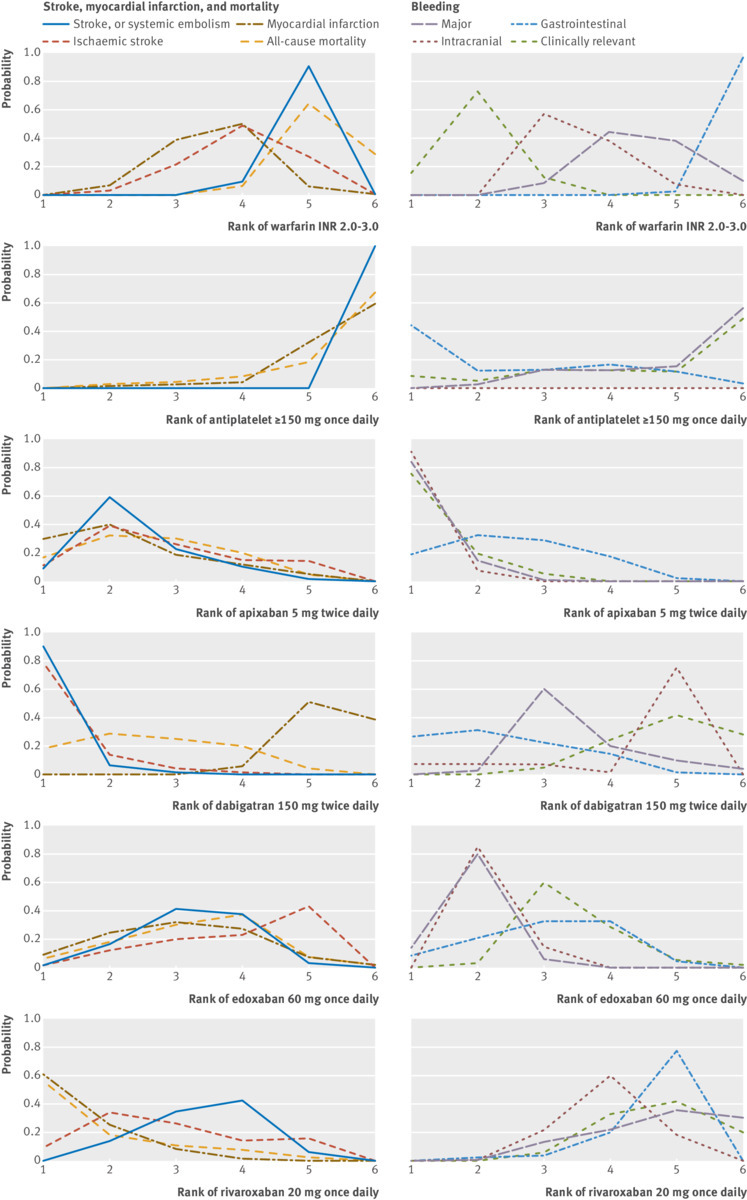
Rankograms for doses of licensed products examined in prevention of stroke in patients with atrial fibrillation

Figure 4 shows that apixaban 5 mg twice daily was ranked as being the most effective intervention for several of the outcomes evaluated including stroke or systemic embolism, myocardial infarction, and all-cause mortality. It was also ranked as being the safest with lowest incidence of major and gastrointestinal bleeding. Edoxaban 60 mg once daily was ranked second for major bleeding and all-cause mortality. Except for the outcome of all-cause mortality, rivaroxaban 20 mg once daily was ranked lowest of the DOACs. The non-DOAC interventions (warfarin dosed to achieve an INR 2.0-3.0 and antiplatelet ≥150 mg once daily) were ranked lowest for stroke or systemic embolism.

### Cost effectiveness results

**Fig 5:**
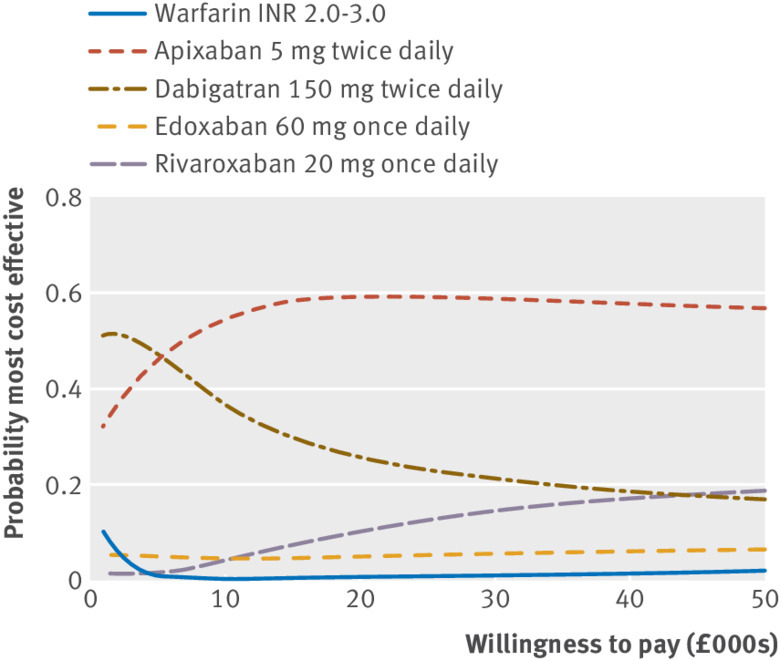
Cost effectiveness acceptability curves. The probability each preferred intervention is most cost effective against willingness to pay for each QALY threshold

Table 4 shows that dabigatran 150 mg twice daily had the lowest expected lifetime total cost (£23 064) for a patient aged 70 starting anticoagulation, followed by apixaban 5 mg twice daily, edoxaban 60 mg once daily, warfarin, and rivaroxaban 20 mg once daily which had the highest expected lifetime total cost (£24 841), although there is substantial uncertainty around these estimates. Apixaban 5 mg twice daily had the highest expected quality adjusted life years (QALYs) (5.49), followed by rivaroxaban 20 mg once daily (5.45), dabigatran 150 mg twice daily (5.42), edoxaban 60 mg once daily (5.41), and warfarin (5.17), though again there is substantial uncertainty. Assuming that the UK NHS is willing to pay £20 000 for each year of perfect health (one QALY), all DOACs have a positive expected incremental net benefit compared with warfarin. Apixaban 5 mg twice daily has the highest expected incremental net benefit (£7533), followed by dabigatran 150 mg twice daily (£6365), rivaroxaban 20 mg once daily (£5279), and edoxaban 60 mg once daily (£5212). Apixaban 5 mg twice daily is the only DOAC for which the 95% confidence interval around incremental net benefit is positive, suggesting that apixaban is cost effective compared with warfarin. Similar results were found for the higher £30 000 threshold. Uncertainty in the estimated total costs and QALYs is illustrated in the cost effectiveness plane (web appendix 7). Figure 5 shows that apixaban 5 mg twice daily has the highest probability of being the most cost effective product for prevention of stroke in patients with atrial fibrillation of the five selected for comparison. It has a probability close to 60% in the £20 000-£30 000 range of willingness to pay, which is the range generally considered by NICE. Warfarin and edoxaban 60 mg twice daily are unlikely to be cost effective. Sensitivity analyses, described in further detail in web appendix 2, found that these results and conclusions were robust to changes in our assumptions. Dabigatran, edoxaban, and rivaroxaban would have to be sold at the negative annual prices of -£280, -£1140, and -£1173, respectively, in order to become more cost effective than apixaban.

**Table 4:** Cost effectiveness of preferred licensed products for prevention of stroke in patients with atrial fibrillation. Expected (mean) values reported (95% confidence intervals). Incremental values are relative to warfarin international normalised ratio (INR) 2.0-3.0

	Warfarin INR 2.0-3.0	Apixaban 5 mg twice daily	Dabigatran 150 mg twice daily	Edoxaban 60 mg once daily	Rivaroxaban 20 mg once daily
Total costs (£)					
Expected	24 418 (12 189 to 50 365)	23 340 (12 842 to 45 753)	23 064 (12 674 to 46 075)	23 985 (13 098 to 46 319)	24 841 (13 198 to 47 603)
Expected incremental	NA	−1078 (−7626 to 2568)	−1354 (−8049 to 2273)	−433 (−6430 to 3619)	422 (−4730 to 5104)
QALYs					
Expected	5.166 (3.629 to 6.541)	5.488 (3.841 to 6.795)	5.416 (3.817 to 6.701)	5.405 (3.819 to 6.678)	5.451 (3.824 to 6.797)
Expected incremental	NA	0.323 (−0.015 to 0.814)	0.251 (−0.080 to 0.703)	0.239 (−0.112 to 0.684)	0.285 (−0.068 to 0.810)
Expected incremental net benefit at threshold (£)					
£20 000	NA	7533 (490 to 18 228)	6365 (−168 to 17 039)	5212 (−894 to 14 826)	5279 (−1097 to 15 180)
£30 000	NA	10 760 (576 to 25 861)	8871 (−597 to 23 402)	7601 (−1556 to 20 987)	8130 (−1399 to 22 819)

QALY=quality adjusted life year; NA=not applicable

## Discussion

To our knowledge, this is the first study of all currently licensed direct acting oral anticoagulants (DOACs) for stroke prevention in patients with atrial fibrillation that has provided a rank order for their use, in terms of both individual efficacy and safety outcomes and overall cost effectiveness. DOACs appear to be at least as effective as warfarin in reducing the risk of stroke secondary to atrial fibrillation. They are associated with a reduced risk of bleeding compared with warfarin at doses to maintain an international normalised ratio (INR) between 2.0 and 3.0, although the risk of bleeding with these drugs is still substantial and some patient populations still require monitoring between DOACs, based on network meta-analyses, suggested that apixaban 5 mg twice daily and edoxaban 60 mg twice daily reduced the risk of major bleeding the most compared with warfarin, while maintaining efficacy in reducing the risk of stroke. This advantage of apixaban offsets its slightly higher cost compared with other DOACs and thus apixaban is associated with the highest net benefit and quality adjusted life years (QALYs) in the cost effectiveness analysis. We found no convincing evidence of effect modification owing to mean time in warfarin therapeutic range, mean age, percentage of male patients, or mean CHADS_2_ score across the different outcomes.

Our study has several implications for clinical practice. Current NICE guidance does not indicate whether one drug with the same mechanism of action should be used over another.^7^ Providing evidence for development of prescribing guidelines is important for rational drug use, and may reduce costs of stocking multiple drugs. Our analysis indicates that, of the currently available DOACs, apixaban ranks highest on the balance of efficacy, safety, and cost. Policy makers, healthcare providers, and patients could therefore consider apixaban to be the first choice among DOACs for the prevention of stroke in most patients with atrial fibrillation, based on currently available evidence. However, clear guidance will be needed on the hierarchy of DOACs for stroke prevention in patients with atrial fibrillation, specifically a treatment hierarchy and conditions under which alternative drugs from within the same class should be prescribed (eg, as reserve treatments for patients with specific contraindications or adverse reactions to apixaban). This approach should increase the use of this drug class, benefit patient safety, and lead to eventual cost savings.

### Strengths and weaknesses in relation to other studies

Our findings about the overall efficacy and safety of DOACs are consistent with previously published meta-analyses and postapproval observational studies.^10^ ^12^ ^34^ ^35^ ^36^ The probabilities used in our cost effectiveness analysis for outcomes including stroke, major bleeding, and all-cause mortality are also comparable to those observed in recent prospective atrial fibrillation registry studies.^37^ A previous review with a similar analysis approach also ranked apixaban 5 mg twice daily and dabigatran 150 mg twice daily as the most cost effective drugs.^38^ However, this previous review only included five trials, hence there was considerable uncertainty around the conclusions and some relevant drug products could not be examined.

Recent studies have suggested that the efficacy and safety of dabigatran could be improved by monitoring the achieved drug levels, because these exhibit wide interpatient variation.^39^ This may reduce the convenience of this DOAC and increase its cost compared with warfarin or other DOACs. A question has also been raised about the efficacy data for rivaroxaban: the largest efficacy study used an INR testing device which gave faulty readings.^40^ This means that a proportion of patients on warfarin may have been underdosed, inflating the relative efficacy of rivaroxaban. However, the number of patients using the INR testing device was low. The FDA have reanalysed the data to assess the impact of the faulty readings on the results and concluded that the effects on stroke or bleeding rates were minimal.^41^

### Strengths and weaknesses of this study

The strengths of our study include comprehensive coverage of current research findings, careful appraisal of study quality, a focus on clinically relevant endpoints, and comprehensive analyses allowing comparisons between DOACs as well as comparisons of DOACs with warfarin. Limitations relate mainly to assumptions underlying the network meta-analysis and to limitations of the primary data. There were no direct comparisons between DOACs, necessitating a network meta-analysis approach. We were unable to fit random effects models because few comparisons were replicated in two or more trials. The network meta-analyses assume that studies making different comparisons do not differ in participant characteristics that are associated with response to treatment (effect modifiers). Where data were available for meta-regression analyses and comparisons of direct versus indirect sources of evidence, we observed no clear evidence of effect modification.

Table 1 shows that many of the outcomes extracted for our review were incompletely reported. Such incomplete reporting reduces precision, and is a threat to the validity of results of systematic reviews if non-reporting of outcomes is influenced by the direction or statistical significance of the intervention effect. However, among the larger phase III trials comparing a DOAC with warfarin (ARISTOTLE, AVERROES, ENGAGE AF-TIMI 48, RE-LY and ROCKET AF) results for the following outcomes were all reported or could be derived from their components: stroke or systemic embolism, ischaemic stroke, myocardial infarction, all-cause mortality, major bleeding, intracranial bleeding, and gastrointestinal bleeding. This gives some reassurance that the conclusions of our review are unlikely to have been substantially affected by bias owing to selective reporting of outcomes. It is nonetheless unfortunate that the outcome of clinically relevant bleeding was not reported by RE-LY. Studies were generally assessed as having a low risk of bias except that most were open label so were assessed as having a high risk of bias owing to a lack of blinding.

Our findings are limited by the constraints of cost effectiveness analyses. These make long term projections on the basis of short term trial evidence, observational data, and clinically informed assumptions about treatment pathways and health state transitions. Furthermore, the profile of patients treated in trials may not be the same as those treated in practice. Older patients and those with multiple comorbidities, who may have a higher risk of bleeding than younger patients with fewer comorbidities, have been excluded from many trials. Finally, the long term safety of DOACS will only emerge as this drug class becomes more widely used in large patient populations in the future.

A head to head trial comparing different DOAC drugs would overcome the need for indirect comparisons to be made through network meta-analysis and improve the precision of estimates of relative efficacy and safety. Our cost effectiveness analyses are sensitive to these indirect comparisons, many of which are not precisely estimated. The analyses are also sensitive to costs, the effect of past events on future hazard ratios, and probabilities of treatment switching. A head to head trial would provide valuable information on these measures, although measuring all outcomes with sufficient precision would require a very large trial, which could be prohibitively expensive. Furthermore, the additional benefits and convenience for older patients and their carers are important factors to be considered.

### Conclusion

DOACs appear to be at least equivalent to warfarin at preventing stroke in patients with atrial fibrillation and to carry a reduced risk of bleeding. They overcome some of the limitations associated with warfarin and may lead to increased use by patients with atrial fibrillation. The cost of anticoagulation may be greatly reduced once generic DOACs become available, since they do not require monitoring, the major cost associated with warfarin use. Despite a similar mechanism of action, apixaban at the right dose appears to maximise efficacy and safety among the DOACs, with favourable cost effectiveness. Further long term data may bring other insights with respect to safety, and it is important to identify patient groups that may not benefit from DOACs, as well as to develop drugs to reverse the anticoagulant effects of each DOAC.^9^ Additional investments in new trials that address limitations of the current evidence may help practitioners and policy makers better understand the role of DOACs in this clinical setting.

**What is already known on this topic**

Anticoagulants have an established role in the prevention of stroke in patients with atrial fibrillation
Direct acting oral anticoagulants (DOACs) overcome some of the limitations of warfarin which include monitoring, slow onset of action, bridging, and multiple drug interactions
Randomised controlled trials, and meta-analyses of these trials, suggest that DOACs, as a class, reduce the risk of stroke or systemic embolic events compared with warfarin, and that they may be safer with respect to the risk of bleeding

**What this study adds**

DOACs, as a class, reduce the risk of stroke and all-cause mortality in patients with atrial fibrillation, and are safer with respect to major and intracranial bleeding than warfarin when used at doses to maintain an international normalised ratio (INR) of 2.0-3.0
Our cost effectiveness analysis provides evidence that, despite their higher costs, several DOACs are preferable to warfarin
We found that apixaban 5 mg twice daily has the highest expected incremental net benefit, followed by rivaroxaban 20 mg once daily, edoxaban 60 mg once daily, and dabigatran 150 mg twice daily

## Web Extra

Extra material supplied by the author

## Notes

Contributors: JACS, RS, and ADH conceived the project. GNO, PAD, PNB, and JS selected articles for inclusion, extracted data, and assessed the risk of bias. JACS, JPTH, JALL, ADH, and RS selected results for inclusion. JACS, JPTH, SD, NJW, JALL, and DMC planned the statistical analyses. JALL performed the network meta-analyses. NJW, WH, HHZT, and PAB developed and analysed the economic models. HHZT performed the cost effectiveness analyses. ADH, RS, and CS provided clinical expertise. DE and ASB provided a patient perspective. PNB provided pharmaceutical expertise. JALL, JPTH, RS, and HHZT wrote the first draft of the paper and all authors revised it critically for important intellectual content. All authors have approved this version for publication. JACS is the study guarantor.

Ethical approval: Not required.

Data sharing: No additional data are available

Transparency: The corresponding author, JACS, affirms that this manuscript is an honest, accurate, and transparent account of the study being reported; that no important aspects of the study have been omitted; and that any discrepancies from the study as planned (and, if relevant, registered) have been explained.
