# Supplementary material for: Oral anticoagulants for prevention of stroke in atrial fibrillation: systematic review, network meta-analysis, and cost effectiveness analysis
Source: BMJ. 2017 Nov 28;359:j5058. doi: 10.1136/bmj.j5058 (PMC5704695; doi:10.1136/bmj.j5058)
Supplement: Supplementary file 1 — Appendix: Supplementary materials [file lopj038668.ww1.pdf]

Appendix 1: Medline search strategy

The search was run on the 20th March 2014 and updated on the 17th August 2016.

Database: Epub Ahead of Print, In-Process & Other Non-Indexed Citations, Ovid MEDLINE(R) Daily and Ovid MEDLINE(R) <1946 to Present>

Search Strategy:

* = new terms added for August 2016 update search.

----

1 tachycardia, supraventricular/ or tachycardia, ectopic atrial/ (6082)

2 atrial fibrillation/ (41512)

3 ((atrial or atrium or auricular) adj3 fibrillat$).ti,ab. (50715)

4 heart fibrillat$.ti,ab. (45)

5 (supraventricul$ adj3 (arrhythmi$ or tachycardia$)).ti,ab. (8252)

6 ((atrial or atrium) adj3 (tachycardia$ or arrhythmi$)).ti,ab. (8031)

7 (atrial adj3 tachyarrhythmi$).ti,ab. (1447)

8 Atrial Flutter/ (5360)

9 ((atrial or auricular) adj3 flutter$).ti,ab. (6064)

10 or/1-9 (74097)

11 exp *Anticoagulants/ (103499)

12 exp *Coumarins/ (28593)

13 Warfarin/ (16524)

14 exp Vitamin K/ai [Antagonists & Inhibitors] (2025)

15 Thrombin/ai [Antagonists & Inhibitors] (3518)

16 Factor Xa/ai [Antagonists & Inhibitors] (0)

17 Aspirin/ (40902)

18 (anticoagula$ or anti-coagula$).ti. (25833)

19 (oral anticoagula$ or oral anti-coagula$).ti,ab. (11048)

20 (coumarin$ or coumadin$ or warfarin or marevan or dicoumarol or dicoumarin or dicumarin or dicumarol or acenocoumarol or phenindione or aldocumar).ti,ab. (32012)

21 (factor Xa adj2 (antagonist$ or inhibitor$)).ti,ab. (1989)

22 Dabigatran/* (1792)

23 exp Factor Xa Inhibitors/* (3797)

24 (factor 10a adj2 (antagonist$ or inhibitor$)).ti,ab. (2)

25 (factor IIa adj2 (antagonist$ or inhibitor$)).ti,ab. (39)

26 ((vitamin K or vitamin-k) adj2 (antagonist$ or inhibitor$)).ti,ab. (3571)

27 (dabigatran or pradaxa or BIBR1048 or Apixaban or Eliquis or BMS-562247-01 or Edoxaban or Lixiana or savaysa or DU-176b or betrixaban or PRT-054021 or PRT0504021 or rivaroxaban or xarelto or BAY-59739 or Erixaban or D0913).ti,ab. (4452)

28 (NOAC or NOACS or DOAC* or DOACS*).ti,ab. (1067)

29 (aspirin or acetyl-salicylic acid or acetylsalicylic acid).ti,ab. (48632)

30 or/11-29 (202351)
31 10 and 30 (10148)
32 letter/ (937091)
33 editorial/ (416013)
34 news/ (179887)
35 exp historical article/ (375007)
36 Anecdotes as topic/ (4729)
37 comment/ (683711)
38 case report/ (1817491)
39 (letter or comment$).ti. (117229)
40 or/32-39 (3729628)
41 randomized controlled trial/ or Randomized Controlled Trials as Topic/ or random$.ti,ab. (1036497)
42 40 not 41 (3695145)
43 animals/ not humans/ (4266992)
44 exp Animals, Laboratory/ (786549)
45 exp Animal Experimentation/ (8140)
46 exp Models, Animal/ (478004)
47 exp rodentia/ (2920238)
48 (rat or rats or mouse or mice).ti. (1217361)
49 or/42-48 (8615045)
50 31 not 49 (7878)
51 meta analysis as topic/ (15295)
52 meta analysis/ (72397)
53 (meta analy$ or metaanaly$ or metanaly$ or meta regression).ti,ab. (102686)
54 ((systematic$ or evidence$) adj2 (review$ or overview$)).ti,ab. (117330)
55 (reference list$ or bibliograph$ or hand search$ or manual search$ or relevant journals).ab. (33934)
56 (search strategy or search criteria or systematic search or study selection or data extraction).ab. (36335)
57 (search$ adj4 literature).ab. (40690)
58 (medline or pubmed or cochrane or embase or psychlit or psyclit or psychinfo or cinahl or science citation index or bids or cancerlit).ab. (131256)
59 cochrane.jw. (16120)
60 ((multiple treatment$ or indirect or mixed) adj2 comparison).ti,ab. (1291)
61 or/51-60 (284947)
62 randomized controlled trial.pt. or randomized controlled trial/ or Randomized Controlled Trials as Topic/ (532167)
63 controlled clinical trial.pt. (91561)
64 randomi#ed.ab. (441404)

65 placebo.ab. (177912)

66 drug therapy.fs. (1897621)

67 randomly.ab. (261690)

68 trial.ab. (381949)

69 groups.ab. (1628894)

70 or/62-69 (3916797)

71 clinical trials as topic.sh. (178961)

72 trial.ti. (160523)

73 or/62-65,67,71-72 (1133412)

74 61 or 73 (1333096)

75 50 and 74 (2508)

76 (201409$ or 201410$ or 201411$ or 201412$ or 2015$ or 2016$).ed,ep,dc. (3065201)

77 75 and 76 (700)

78 limit 75 to yr="2015 -Current" (458)

79 77 or 78 (700)
