# Supplementary material for: Oral anticoagulants for prevention of stroke in atrial fibrillation: systematic review, network meta-analysis, and cost effectiveness analysis
Source: BMJ. 2017 Nov 28;359:j5058. doi: 10.1136/bmj.j5058 (PMC5704695; doi:10.1136/bmj.j5058)
Supplement: Supplementary file 2 — Appendix: Supplementary materials [file lopj038668.ww2.pdf]

Appendix 2: Main assumptions and structure of the economic model

We assumed that before the introduction of DOACs standard of care for AF patients was warfarin. For patients on warfarin as first-line treatment, the second-line intervention was assumed to be no treatment. For patients on a DOAC as first-line treatment, second line treatment may be either warfarin or no treatment, depending on the reason for discontinuation. No treatment is the only third-line option available.

Each treatment strategy has the same model structure but with different costs, utilities, and event probabilities. From any state, a patient can have a clinically relevant (extracranial) bleed, an intracranial haemorrhage (ICH), an ischaemic stroke or a myocardial infarction (MI), all of which have long-term consequences that are modelled. Patients can also experience transient ischaemic attack (TIA) or systemic embolism (SE), which are transient events, or can discontinue or switch treatment due to any events, or die. Patients are assumed to always switch treatment from dabigatran to warfarin if they experience an MI, due to recent findings suggesting a link between dabigatran and MI risk (1). However, following ischemic stroke, TIA or SE the probability of switching is only 10% so most patients do not discontinue. The probability of switching is also only 30% following a clinically relevant bleed.

Transition probabilities were derived using hazard ratios for the DOACs estimated from the systematic literature review. The warfarin arms were used to estimate baseline hazards. We relied on previous meta-analyses to estimate the relative effect of warfarin compared to no treatment.(2) Evidence from the literature was used to estimate the effect of prior events on the future risk of stroke, mortality, MI, SE, TIA, and bleed risk.(3, 4) No evidence was available for the effect of prior bleeds or ICH on mortality so we assumed history of bleeds or ICH would have the same effect on future risk of death as history of stroke. Our model makes the simplifying assumption that SE and TIA are transient events with no long term impact on costs, quality of life or future risks. It is likely that a history of SE and TIA may have such an impact but evidence on this was limited and impact was expected to be minimal by our clinicians. Our assumption that baseline and relative effects are independent of age is a limitation as comorbidities (eg. chronic kidney disease) become more common with age, although this does not necessarily impact relative effects.

Average drug costs were based on the BNF March 2015 update.(5) As all the DOACs are taken orally it was assumed that there are no administration or monitoring costs.(6) Average drug and monitoring cost of warfarin comes from a costing report by NICE.(7) Acute management costs for SE, MI, TIA, and clinically relevant bleeding come from the 2013/14 NHS reference costs.(8) These events were assumed to have no long term management costs. Acute and long term management costs for ischaemic stroke and ICH came from a study of AF patients on a UK stroke registry.(9) Instantaneous event disutilities and long-term quality of life consequences were identified from a previous NICE technology appraisal submission on rivaroxaban,(10) which conducted a systematic literature search for evidence on EQ-5D utility index in health states related to AF.

As our model makes a range of assumptions, sensitivity analyses were important. . Several sensitivity analyses were conducted to test the robustness of the analysis to our assumptions: (1) setting warfarin monitoring and administration costs to zero; (2) assuming ICH and bleed had the no effect on future mortality, unlike our base case which assumed the same effect as a history of stroke; (3) assuming patients only switched to no treatment following an ICH or MI (if on dabigatran); (4) assuming patients switched after stroke, bleed, ICH or (if on dabigatran) MI; (5) removing the BAATAF study, in which control patients could receive aspirin, from the meta-analysis comparing warfarin to no treatment; (6) assuming that lower doses of apixaban (2.5mg) and dabigatran (110mg) are used in older patients (as for the base-case doses, efficacy and safety rates for the lower doses are also taken from our NMA); (7) setting the hazard ratio for ICH on warfarin relative to no treatment to 1, as an alternative to the base case assumption that it was the same as that for bleed (0.51 (0.205, 0.949)); (8) including long-term management costs for MI of £142 per year, in line with the Bayer submission on rivaroxaban.(10); (9) analysing a cohort with initial age 60, which affects utilities and mortality in the model but costs and other event rates were independent of age and remained the same as the base case; (10) as for (9), but with initial age 80.

The conclusion that apixaban 5mg is most cost-effective was robust to all of our sensitivity analyses except for sensitivity analysis (4) where warfarin became most cost-effective, due to patients spending less time on a DOAC because of treatment switching). For sensitivity analysis (6) but apixaban (with age-specific dose) was most cost-effective, although there was greater uncertainty.

As dabigatran and other DOACs are likely to fall in price when they come off patent, we performed threshold analyses to calculate the price at which DOACs would have to be sold for their expected incremental net benefit to becomes the maximum; in other words, the price at which they become most cost-effective. We found that, to be more cost-effective than apixaban, dabigatran's price would have to reduce from an annual cost of £801.76 to -£280.35, edoxaban would have to reduce to -£1140.17, and rivaroxaban would have to reduce from £766.52 to -£1172.97. In other words, they are unlikely to become more cost-effective than apixaban at any non-negative price.

A2.1: Main model assumptions

Does not include minor non-clinically relevant bleeds as transient events.
No distinction between severity of ischaemic strokes.
Dose of apixaban and dabigatran given does not reduce as patients age.
Bleeds and ICH (and with it, haemorrhagic stroke) have same effect on future risk of death as stroke
Patients on dabigatran who experience an MI will always switch to warfarin.
Patients switch to no treatment after ICH/haemorrhagic stroke.

Patients may switch (with an assumed probability) from DOAC to warfarin or warfarin to no treatment after ischaemic stroke, bleed, SE or TIA.
Patients may (with an assumed probability) discontinue warfarin treatment or switch from a DOAC to warfarin, even if they do not experience an event (due to lack of compliance).
Warfarin arms from the RCTs identified in our systematic review are representative of the AF population in England and Wales.
Events rate and relative treatment effects are assumed not to vary with age.
Relative mortality rate in AF patients relative to the general population does not vary with age.
Warfarin treatment costs over 3 months are taken from the NICE costing report. Uncertainty in this is represented using a uniformly distribution from 50% to 150% of the NICE costing report estimate.
Assumes no monitoring or administration costs for DOACs
Assumes post-ICH management costs to be similar to post-ischaemic stroke management costs.
Combined management costs for post-multiple event states (eg. MI+Stroke) to be the maximum of management costs for constituent events.
Assumed quality of life for patients with a history of multiple events to be multiplicative combination of quality of life for constituent events.

A2.2: Illustration of the Markov model

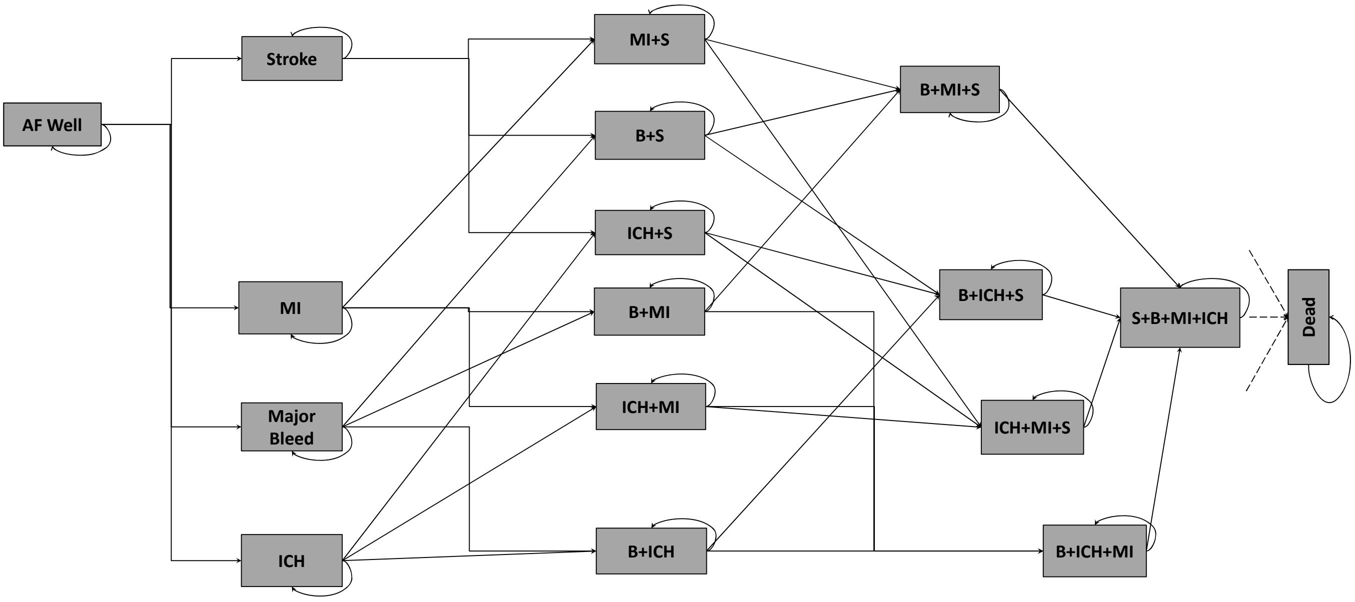

* Patients can experience transient events (TIA or SE) but stay in same health state, with possibly changed treatment, thereafter. (S = ischaemic stroke, B = other clinically relevant bleed, ICH = intra-cranial haemorrhage, MI = myocardial infarction)

A2.3: Summary of estimates of treatment switching probabilities, costs and utilities used in the economic model

Item	Mean (or reported values from source)	Distribution****	Source
**Treatment switching probabilities per 3 month cycle***********			
Following stroke or bleed	0.30 (CrI 0.00-1.00)	Beta(0.3, 0.7)	Assumption
Following SE or TIA	0.10 (CrI 0.00-1.00)	Beta(0.1, 0.9)	Assumption
**Drug and administration costs per 3 month cycle**			
Apixaban 10mg	£200.42	Fixed	BNF(11) and ONS(12)
Apixaban 5mg	£200.44	Fixed	
Dabigatran 150mg	£200.44	Fixed	
Dabigatran 110mg	£200.44	Fixed	
Rivaroxaban 20mg	£191.63	Fixed	
Edoxaban 60mg	£200.44	Fixed	Not in BNF 2013, assumed same as Apixaban 5mg
Warfarin	£101.13	Uniform(52.57, 157.70)	National Institute for Health and Clinical Excellence (NICE), Costing Report: Implementing NICE Guidance in England. Atrial Fibrillation: The Management of Atrial Fibrillation. NICE Clinical Guideline, 2006. 36.(7)*
**Acute event costs**			
Ischaemic stroke	11626 (SD=16868)	Normal (11626, 1325)	Ischaemic stroke, all strokes.(9)**
ICH	11453 (SD=13815)	Normal (11453, 3350)	ICH or haemorrhagic stroke, all haemorrhagic strokes.(9)
SE (non-fatal)	2373	Uniform (1186.5, 3559.5)	NHS reference costs.(8)
TIA	1064	Uniform (532, 1596)	NHS reference costs.(8)
Clinically relevant bleeding***	1751.5	Uniform (875.75, 2627.25)	NHS reference costs.(8)
MI	4830	Uniform (2415.24, 7245.72)	Acute MI, NHS reference costs for hospitalization,(8) doubled to include follow-up costs
**Annual post-ischaemic stroke and post-ICH management costs. These are divided by four to obtain 3-monthly cycle costs. Only average cost used in model.**			
Non-disabling	2135 (SD=3676, n=66)		Luengo et al.(9)

Moderately disabling	4165 (SD=7668, n=58)		Luengo et al.(9)
Totally disabling	6324 (SD=14898, n=6324)		Luengo et al.(9)
Average (Ischaemic stroke and ICH)	3613 (SD=4235, n=136)	Normal(3613, 363)	Weighted average of the mean and SDs reported in Luengo et al,(9) inflated to 2013/14
**Reference group health utilities**			
Stable AF quality of life (for AF model)	0.779 (SD=0.253, n=3045, SE=0.0045)	Normal(0.779, 0.0045)	Berg 2010(13)
**Acute health event disutilities*******			
TIA and SE disutility	-0.131	Uniform(-0.197, -0.066)	Robinson 2001.(14)
Acute Ischaemic stroke disutility	-0.59	Uniform(-0.885, -0.295)	Robinson 2001.(14)
Acute ICH disutility	Median utility 0.60 (95% CI 0.02-1.00) (n=60)	Normal(0.60, 0.064) – AF well	Lenert 1997.(15)
Other CRB disutility	-0.03 (SE=0.001531)	Normal(-0.03, 0.001531)	Robinson 2001.(14)
Acute MI disutility	0.683 utility (SD=0.233, n=222, SE=0.0156)	Normal(0.683, 0.0156) – AF well	Lacey 2003.(16)******
**Chronic health state annual quality of life**			
Post Ischaemic stroke quality of life	0.69 (SD=0.18, n=77, SE=0.0205)	Normal(0.69, 0.0205)	Haacke 2006.(17) *******
Post ICH quality of life	0.74 (SD=0.39, n=5, SE=0.1744)	Beta(3.941, 1.385)	Haacke 2006.(17) ********
Post MI quality of life	0.718 (SD=0.243, n=222, SE=0.0163)	Normal(0.718, 0.0163)	Lacey 2003.(16)******

* We inflated to 2013/14 values using the ONS Consumer Price Inflation index for medical services (DKC3)(12) and placed a Uniform distribution ~(52.57, 157.70) and (210.26, 630.79) (on the cost per three month and yearly cycles respectively.
** We inflated to 2013/14 values using the ONS Consumer Price Inflation index for medical services (DKC3).(12)
*** Average of gastrointestinal and non-gastrointestinal bleed
**** Capped above at 1 for quality of life and 0 for disutility
***** Disutilities assumed to last for 3 months.
****** Table 3, year mean EQ-5D score
******* Table 2 in source article, weighted average EQ-5D score for ischaemic stroke
******** Table 3 in source article, EQ-5D for haemorrhagic stroke.
********* Following stroke, bleed, SE or TIA, patients may switch from DOAC to warfarin or from warfarin to no treatment with the specified probabilities.

A2.4: Hazards and hazard ratios used in the economic model not from base case NMA.

	Ischaemic stroke	TIA	Systemic Embolism*	Intracranial Haemorrhage	Clinically relevant bleeding	MI	Death (all causes)

Warfarin Baseline hazards	0.012 (0.01, 0.013)	0.025 (0.006, 0.089)	0.017 (0.0059, 0.041)	0.0094 (0.0057, 0.017)	0.066 (0.031, 0.13)	0.0079 (0.0064, 0.01)	0.038 (0.028, 0.052)
No treatment hazard ratio**	3 (1.84, 4.83)	2.69 (0.0659, 9.94)	19.2 (0.085, 39.4)	NA***	0.51 (0.205, 0.949)	NA****	1.65 (0.575, 3.57)

*Systemic embolism excludes stroke events

** Random effects meta-analyses of each outcome using 6 studies reported by Hart et al.(2) No treatment was mixed with placebo. Note that in one study (BAATAF) control patients could choose to take Aspirin. No evidence for MI and insufficient evidence for ICH.

*** Hazard ratio assumed the same as for bleed and set to 1 in sensitivity analysis.

**** No effect of warfarin assumed for MI

A2.5: Estimated log-hazard ratio (standard error) for the effect of previous events on future events.(3,4)

Risk factor	Future ischaemic stroke	Future TIA/SE	Future ICH	Future Bleed	Future Death
Stroke	1.39 (0.03)	1.28 (0.02)	0.49 (0.09)	0.33 (0.05)	0.28 (0.15)
ICH	0.58 (0.07)	0.60 (0.06)	2.32 (0.09)	1.08 (0.07)	0.28 (0.15)
Bleed	0.28 (0.04)	0.31 (0.04)	1.26 (0.08)	1.20 (0.04)	0.28 (0.15)
MI	0.22 (0.03)	0.25 (0.03)	-0.06 (0.09)	0.22 (0.04)	0.03 (0.18)

*Normal distributions are used to reflect uncertainty in the estimated log-hazard ratios.

Additional references in Appendix 2

1. Hori M, Connolly SJ, Zhu J, et al. Dabigatran Versus Warfarin: Effects on Ischemic and Hemorrhagic Strokes and Bleeding in Asians and Non-Asians With Atrial Fibrillation. Stroke 2013;44(7):1891-6.

2. Hart RG, Pearce LA, Aguilar MI. Meta-analysis: Antithrombotic Therapy to Prevent Stroke in Patients Who Have Nonvalvular Atrial Fibrillation. Ann Intern Med 2007;146(12): 857-67.

3. Friberg L, Rosenqvist M, Lip GYH. Evaluation of risk stratification schemes for ischaemic stroke and bleeding in 182678 patients with atrial fibrillation: the Swedish Atrial Fibrillation cohort study. European Heart Journal 2012;33(12):1500-10.

4. Andersen KK, Olsen TS. Reduced Poststroke Mortality in Patients With Stroke and Atrial Fibrillation Treated With Anticoagulants: Results From a Danish Quality-Control Registry of 22179 Patients With Ischemic Stroke. Stroke 2007;38(2):259-63.

5. Gomez-Outes A, Terleira-Fernandez AI, Suarez-Gea ML, Vargas-Castrillon E. Dabigatran, rivaroxaban, or apixaban versus enoxaparin for thromboprophylaxis after total hip or knee replacement: systematic review, meta-analysis, and indirect treatment comparisons. BMJ 2012;344:e3675.

6. Ali A, Bailey C, Abdelhafiz AH. Stroke prophylaxis with warfarin or dabigatran for patients with non-valvular atrial fibrillation-cost analysis. Age and Ageing 2012;41(5):681-4.

7. National Institute for Health and Clinical Excellence (NICE). Costing Report: Implementing NICE Guidance in England. Atrial Fibrillation: The Management fo Atrial Fibrillation. NICE Clinical Guideline 2006;36.

8. Department of Health. NHS Reference Costs 2012-13. London, UK; 2014.
9. Luengo-Fernandez R, Yiin GSC, Gray AM, Rothwell PM. Population-based study of actue- and long-term care costs after stroke in patients with AF. International Journal of Stroke 2013; 8(5): 308-14.

10. Bayer Plc. Single Technology Appraisal (STA) of Rivaroxaban (Xarelto). STA 2011.

11. BNF, British National Formulary, B.G.a.P. Press, Editor. 2013: London.

12. Office for National Statistics (ONS). ONS Consumer Price Inflation index for medical services (DKC3) for 2013/14. 2013 November 1 2015]; Available from: http://www.ons.gov.uk/ons/publications/re-reference-tables.html?edition=tcm%3A77-323625,%202015%20#29

13. Berg J, Lindgren P, Nieuwlaat R, Bouin O, Crijns H. Factors determining utility measured with the EQ-5D in patients with atrial fibrillation. Qual Life Res 2010;19(3):381-390.

14. Robinson A, Thomson R, Parkin D, Sudlow M, Eccles M. How patients with atrial fibrillation value different health outcomes: a standard gamble study. J Health Serv Res Policy 2001;6(2):92-8.

15. Lenert L, Soetikno RM. Automated Computer Interviews to Elicit Utilities: Potential Applications in the Treatment of Deep Venous Thrombosis. JAMA 1997; 4(1):49-56.

16. Lacey E, Walters SJ. Continuing inequality: gender and social class influences on self perceived health after a heart attack. J Epidemiol Community Health 2003;57(8):622-627.

17. Haacke C, Althaus A, Spottke A, Siebert U, Back T, Dodel R. Long-Term Outcome After Stroke: Evaluating Health-Related Quality of Life Using Utility Measurements. Stroke 2006;37(1):193-198.
