# Supplementary material for: Oral anticoagulants for prevention of stroke in atrial fibrillation: systematic review, network meta-analysis, and cost effectiveness analysis
Source: BMJ. 2017 Nov 28;359:j5058. doi: 10.1136/bmj.j5058 (PMC5704695; doi:10.1136/bmj.j5058)
Supplement: Supplementary file 3 — Appendix: Supplementary materials [file lopj038668.ww3.pdf]

Appendix 3: Characteristics of 23 included randomised trials in stroke prevention in AF (references below)

Study<br><br>(Centre type)<br>[Countries]	Study type<br><br>Sponsor<br>(sponsor's role)	Age eligibility (Mean age)<br>[% Male]	AF type	No. rand.	Interventions compared	Tmt duration (months)	Mean time in therapeutic range (INR)	Outcomes	Time of outcome assessment (months)
**ACTIVE W(1)**<br><br>(Multicentre)<br><br>[North & South America, Europe, Russia, Israel, Australia, Asia, South Africa]	Phase III<br><br>Sanofi -Aventis and Bristol-Myers Squibb<br>(The sponsor contributed to the study design "but had no role in data collection, data analysis, data Interpretation, or writing of the report")	≥18 yrs. (70.2 yrs.)<br><br>[66.1%]	Non-valvular<br><br>ECG diagnosed	6706	**Antiplatelet**<br>1. Clopidogrel 75mg + (aspirin 75-100mg) od<br><br>**Warfarin**<br>2. INR 2-3 (some patients may have received other vitamin K antagonists in use in their country)	Not given	 <br><br><br>63.8%	Efficacy-All stroke, ischemic stroke, haemorrhagic stroke, MI<br><br>Safety-All bleeding, major bleeding, minor bleeding, fatal bleeding, death (all causes)	15.4
**AFASAK(2)**<br><br>(Two centres)<br><br>[Denmark]	Phase III<br><br>NycoMed AS, Oslo, Norway; Henrik Henriksen's Foundation; Kathrine and Vigo Skovgaard's Foundation; and Danish Medical Research Foundation<br>(Not stated)	≥18 yrs. (74.2 yrs.)<br><br>[53.6%]	Chronic non-valvular<br><br>ECG diagnosed	1007	**Warfarin**<br>1. INR 2-3<br><br>**Antiplatelet (aspirin)**<br>2. 75mg od<br><br>3. Placebo od	24	73%	Efficacy-All stroke, fatal stroke, minor ischemic stroke, TIA<br><br>Safety-Bleeding, death (all causes)	24

**AFASAK II(3)**<br><br>(Single centre)<br><br>[Denmark]	Phase III<br><br>The Danish Heart Foundation, Copenhagen; Nycomed DAK A/S Roskilde, Denmark; Du Pont Pharma, Wilmington, Del; The Danish Foundation for Medical Research for the Region of Copenhagen; and many other non-industry funders<br>(Not stated)	≥18 yrs.<br>(74.2 yrs.)<br><br>[60%]	Chronic non-valvular<br><br>ECG diagnosed	677	**Warfarin**<br>1. 1.25mg/day fixed dose<br>2. 1.25mg/day fixed dose plus aspirin 300mg/day od<br>3. INR 2-3<br><br>**Aspirin**<br>4. 300mg od	42	73%	Efficacy-All stroke, ischemic stroke, haemorrhagic stroke, fatal stroke, stroke or systemic embolism, TIA, MI<br><br>Safety-Major bleeding, minor bleeding, intracranial bleeding, death (all causes)	42
**AF-ASA-VKA-CHINA(4)**<br><br>(Two centres)<br><br>[China]	Phase III<br><br>Grant from talent pool subject of Shanghai Shi Dong Hospital<br>(Not applicable)	≥80 yrs.<br>(NR)<br><br>[NR]	Persistent & Permanent non-valvular<br><br>Confirmed by the case history & ECG	110	**Warfarin**<br>1. INR 1.6-2.5<br><br>**Antiplatelet (aspirin)**<br>2. 100mg od	24	NR	Efficacy-Stroke or systemic embolism, ischemic stroke, MI<br><br>Safety-All bleeding, major bleeding, minor bleeding, fatal bleeding, death (all causes)	1, 6, 12, 18, and 24

**AF-DABIG-VKA-JAPAN(5)**<br><br>(Multicentre)<br><br>[Japan]	Phase II<br><br>Boehringer Ingelheim<br>(The sponsor was involved in the trial)	≥20 yrs. (NR)<br><br>[NR]	Paroxysmal, persistent or permanent non-valvular<br><br>ECG diagnosed	174	**Dabigatran**<br>1. 110mg bd<br>2. 150mg bd<br><br>**Warfarin**<br>3. INR 2-3 (INR ≥1.6 to ≤2.6 in ≥70 yrs.)	3	NR	Efficacy-Stroke or systemic embolism<br><br>Safety-All bleeding, major bleeding, composite clinically relevant bleeding	3
**AF-EDOX-VKA-ASIA(6)**<br><br>(Multicentre)<br><br>[Taiwan, South Korea, Hong Kong & Singapore]	Phase II<br><br>Daiichi Sankyo Co., Ltd., Tokyo, Japan<br>(The sponsor had influence on the study design, data management & analysis, and key decisions)	18-80 yrs. (65.1 yrs.)<br><br>[65.4%]	Non-valvular<br><br>ECG diagnosed<br><br>$CHADS_2$≥ 1	235	**Edoxaban**<br>1. 30mg od<br>2. 60mg od<br><br>**Warfarin**<br>3. INR 2-3	3 (Edoxaban)<br><br>6 (Warfarin)	45.1%	Efficacy-Stroke or systemic embolism<br><br>Safety-All bleeding, major bleeding, minor bleeding, clinically relevant non-major bleeding	3
**AF-EDOX-VKA-JAPAN(7)**<br><br>(Multicentre)<br><br>[Japan]	Phase II<br><br>Daiichi Sankyo Co., Ltd., Tokyo, Japan<br>(The funder "had input on the study design and data analysis & interpretation of results and wrote the clinical study report")	≥20 yrs. (NR)<br><br>[NR]	Non-valvular<br><br>ECG diagnosed<br><br>$CHADS_2$≥ 1	536	**Edoxaban**<br>1. 30mg od<br>2. 45mg od<br>3. 60mg od<br><br>**Warfarin**<br>4. INR 2-3 (INR 1.6-2.6 in ≥70 yrs.)	3	83% (≥70 yrs.)<br>73% (<70 yrs.)	Efficacy-Stroke or systemic embolism<br><br>Safety-All bleeding, major bleeding, clinically relevant non-major bleeding, composite clinically relevant bleeding	3

**AF-EDOX-VKA-MULTI(8)**<br><br>(Multicentre)<br><br>[North America, Chile, Europe & Russia]	Phase II<br><br>Daiichi Sankyo Co., Ltd., Tokyo, Japan<br>(Not clear)	18-85 yrs. (65.1 yrs.)<br><br>[62.1%]	Persistent non-valvular<br><br>ECG diagnosed<br><br>$CHADS_2$ ≤2	1146	**Edoxaban**<br>1. 30mg od<br>2. 60mg od<br>3. 30mg bd<br>4. 60mg bd<br><br>**Warfarin**<br>5. INR 2-3	3	49.7%	Efficacy-Stroke or systemic embolism, MI, hospital admission<br><br>Safety-All bleeding, major bleeding, minor bleeding, clinically relevant non-major Bleed, composite clinically relevant bleeding, death (cardiovascular)	3
**AF-VKA-ASA-CHINA(9)**<br><br>(Multicentre)<br><br>[China]	Phase III<br><br>10th National Five-year Project of China<br>(Not applicable)	50-80 yrs. (NR)<br><br>[NR]	Non-valvular<br><br>Diagnosis based on medical history, ECG and/or Holter recordings	690	**Warfarin**<br>1. INR 2.1-2.5<br>2. INR 1.6-2<br><br>**Antiplatelet (aspirin)**<br>3. 200mg od	24 (mean 15)	NR	Efficacy-All stroke, ischemic stroke, haemorrhagic stroke, TIA<br><br>Safety-Major bleeding, minor bleeding, death (all causes)	24

**ARISTOTLE(10-20)**<br><br>(Multicentre)<br><br>[North & South America, Europe, Russia, Israel, Australia, Asia, South Africa]	Phase III<br><br>Bristol-Myers Squibb and Pfizer (The trial was designed in conjunction with the sponsors & "The primary analyses were performed both at Bristol-Myers Squibb and at the Duke Clinical Research Institute")	≥18 yrs. (Median 70 yrs.)<br><br>[64.7%]	Non-valvular or flutter<br><br>ECG diagnosed	18201	**Apixaban**<br>1. 5mg bd<br><br>**Warfarin**<br>2. INR 2-3	21.6 (median)	62.2%	Efficacy-All stroke, ischemic stroke, haemorrhagic stroke, stroke or systemic embolism, MI<br><br>Safety-All bleeding, major bleeding, composite clinically relevant bleeding, intracranial bleeding, death (all causes)	21.6<br><br>(median for Intracranial bleeding)
**ARISTOTLE-J(21)**<br><br>(Multicentre)<br><br>[Japan]	Phase II<br><br>Pfizer Inc. and Bristol-Myers Squibb (Not clear)	≥20 yrs. (70.3 yrs.)<br><br>[82.9%]	Non-valvular<br><br>Diagnosis based on ECG, Holter recording or intracardiac electrogram	222	**Apixaban**<br>1. 2.5mg bd<br>2. 5mg bd<br><br>**Warfarin**<br>3. INR 2-3<br>(INR 2-2.6 in ≥70 yrs.)	3	60%	Efficacy-Stroke or systemic embolism, ischaemic stroke, TIA<br><br>Safety-All bleeding, major bleeding, minor bleeding, clinically relevant non-major bleeding, composite clinically relevant bleeding, death (all causes)	3

**AVERROES(22-25)**<br><br>(Multicentre)<br><br>[North & South America, Europe, Russia, Israel, Australia, Asia, South Africa]	Phase III<br><br>Bristol-Myers Squibb and Pfizer (The sponsor was involved in the design, data collection, and analysis)	≥50 yrs. (70 yrs.)<br><br>[58.5%]	Non-valvular<br><br>ECG diagnosed	5599	**Apixaban**<br>1. 5mg bd (2.5mg if >80 yrs./≤60 kg/renal status)<br><br>**Antiplatelet (aspirin)**<br>2. 81-324mg od	13.1 (mean)		Efficacy-All stroke, stroke or systemic embolism, ischaemic stroke, haemorrhagic stroke, MI<br><br>Safety-Major bleeding, minor bleeding, clinically relevant non-major bleeding, intracranial bleeding, fatal bleeding, death (cardiovascular), death (all causes)	13.1 (mean)
**BAFTA(26)**<br><br>(Multicentre)<br><br>[UK]	Phase III<br><br>The Medical Research Council UK and supported by MidReC and the Primary Care Research trust (The sponsor had no direct role in study design, in data collection, analysis or interpretation, in writing the report, or in the decision to submit for publication)	≥75 yrs. (81.5 yrs.)<br><br>[54.6%]	Non-valvular or atrial flutter<br><br>ECG diagnosed	973	**Antiplatelet (aspirin)**<br>1. 75mg od<br><br>**Warfarin**<br>2. INR 2-3	32.4 (mean)	 <br><br><br><br>67%	Efficacy-All stroke, MI<br><br>Safety-Major bleeding, death (all causes)	32.4 (mean)

**Chinese ATAFS(27)**<br><br>(Multicentre)<br><br>[China]	Phase III<br><br>Not disclosed	40-80 yrs.<br>(63.3 yrs.)<br><br>[59.7%]	Non-valvular	704	**Antiplatelet (aspirin)**<br>1. 150-160mg od<br><br>**Warfarin**<br>2. INR 2-3 (INR 1.6-2.5 in >75 yrs.)	Not reported	NR	Efficacy-All stroke<br><br>Safety-Death (all causes)	2-24<br>(median=19)
**ENGAGE AF-TIMI 48(28, 29)**<br><br>(Multicentre)<br><br>[North & South America, Europe, Russia, Israel, Australia, Asia, South Africa]	Phase III<br><br>Daiichi Sankyo Pharma Development (Not clear)	≥21 yrs.<br>(NR)<br><br>[61.9%]	Non-valvular<br><br>ECG diagnosed<br><br>$CHADS_2$ ≥2	2110<br>5	**Edoxaban**<br>1. 30mg od<br>2. 60mg od<br><br>**Warfarin**<br>3. INR 2-3	29.8<br>(median)	64.9%	Efficacy-All stroke, ischemic stroke, haemorrhagic stroke, fatal stroke, stroke or systemic embolism, MI<br><br>Safety-Major bleeding, minor bleeding, fatal bleeding, intracranial bleeding, clinically relevant non-major bleeding, composite clinically relevant bleeding, death (cardiovascular), death (all causes)	29.8<br>(median)

**EXPLORE-Xa(30)**<br><br>(Multicentre)<br><br>[USA, Canada & Germany]	Phase II<br><br>Portola Pharmaceuticals, South San Francisco, CA, USA<br>(Not stated)	≥18 yrs.<br>(73 yrs.)<br><br>[66.5%]	New or existing non-valvular or atrial flutter<br><br>Diagnosed by Holter, ECG, rhythm strip, pacemaker, or other intracardiac recording	508	**Betrixaban**<br>1. 40mg od<br>2. 60mg od<br>3. 80mg od<br><br>**Warfarin**<br>4. INR 2-3	4.9<br>(mean)	63.4%	Efficacy-All stroke<br><br>Safety-All bleeding, major bleeding, minor bleeding, clinically relevant non-major bleeding, composite clinically relevant bleeding, death (all causes)	4.9<br>(mean)
**J-ROCKET AF(31)**<br><br>(Multicentre)<br><br>[Japan]	Phase III<br><br>Bayer Yakuhin Ltd (The funder was "responsible for trial design and study data collection")	≥20 yrs.<br>(71.1 yrs.)<br><br>[80.6%]	Non-valvular<br><br>ECG diagnosed	1280	**Rivaroxaban**<br>1. 15mg od<br><br>**Warfarin**<br>2. INR 2-3<br>(INR 1.6-2.6 in ≥70 yrs.)	30	65%	Efficacy-All stroke, ischemic stroke, haemorrhagic stroke, stroke or systemic embolism, MI<br><br>Safety-Composite clinically relevant bleeding, death (cardiovascular), death (all causes)	30

**PATAF(32)**<br><br>(Multicentre)<br><br>[Netherlands]	Phase III<br><br>Prevention fund (grant 002817010), Zorg Onder-zoek Nederland; Roche Nicholas BV, Bladel, Holland, donated aspirin (Not stated)	≥60 yrs. (74.8 yrs.)<br><br>[44.9%]	Chronic or intermittent<br><br>ECG diagnosed	729	**Warfarin**<br>1. INR <2<br>2. INR 2.5-3.5 (some patients received other coumarins – phenprocoumon or acenocoumarol)<br><br>**Antiplatelet (aspirin)**<br>3. 150mg od	32.4 (mean)	NR	Efficacy-All stroke, ischaemic stroke, arterial event<br><br>Safety-Death (cardiovascular), death (all causes)	32.4 (mean)
**PETRO(33)**<br><br>(Multicentre)<br><br>[USA, Denmark, Netherlands & Sweden]	Phase II<br><br>Boehringer Ingelheim Pharmaceuticals, Biberach, Germany (The funder was responsible for the statistical analysis conducted according to a prospectively designed plan approved by the steering committee)	≥18 yrs. (69.5 yrs.)<br><br>[81.9%]	Permanent, persistent, & paroxysmal non-valvular with coronary artery disease<br><br>Diagnosis not explained	502*	**Dabigatran**<br>1. 50mg bd<br>2. 50mg + (aspirin 81mg) bd<br>3. 50mg + (aspirin 325mg) bd<br><br>4. 150mg bd<br>5. 150mg + (aspirin 81mg) bd<br>6. 150mg + (aspirin 325mg) bd<br><br>7. 300mg bd<br>8. 300mg + (aspirin 81mg) bd<br>9. 300mg + (aspirin 325mg) bd<br><br>**Warfarin**<br>10. INR 2-3	3	57.2%	Efficacy-Stroke or Systemic embolism<br><br>Safety-All bleeding, major bleeding, composite clinically relevant bleeding	3

**RE-LY(34, 35)**<br><br>(Multicentre)<br><br>[North & South America, Europe, Russia, Israel, Australia, Asia, South Africa]	Phase III<br><br>Boehringer Ingelheim<br>(The sponsor contributed in the design, conduct, and reporting of the study)	≥18 yrs.<br>(71 yrs.)<br><br>[63.6%]	Non-valvular<br><br>ECG diagnosed<br><br>Mean $CHADS_2$ 2.1	18113	**Dabigatran**<br>1. 110mg bd<br>2. 150mg bd<br><br>**Warfarin**<br>3. INR 2-3	24<br>(mean)	64%	Efficacy-Stroke or systemic embolism, ischaemic stroke, haemorrhagic stroke, MI, PE, Hospital admission<br><br>Safety-Major bleeding, minor bleeding, intracranial bleeding, extra-cranial minor bleeding, death (cardiovascular), death (all causes)	24<br>(mean)
**ROCKET AF(36-39)**<br><br>(Multicentre)<br><br>[North & South America, Europe, Russia, Israel, Australia, New Zealand, Asia, South Africa]	Phase III<br><br>Johnson & Johnson and Bayer<br>(The sponsor was not involved in the coordination of the trial, data management, and analyses)	≥18 yrs.<br>(Median 73 yrs.)<br><br>[60.3%]	Non-valvular<br><br>ECG diagnosed<br><br>$CHADS_2$ ≥2	14264	**Rivaroxaban**<br>1. 20mg od<br><br>**Warfarin**<br>2. INR 2-3	19.4<br>(median)	55%	Efficacy-All stroke, stroke or systemic embolism, MI<br><br>Safety-Major bleeding, clinically relevant non-major bleeding, composite clinically relevant bleeding, fatal bleeding, intracranial bleeding, death (all causes)	19.4<br>(median)

**SPAF II(40)**<br><br>(Multicentre)<br><br>[USA]	Phase III<br><br>The Division of Stroke and Trauma, National Institute of Neurological Disorders and Stroke<br>(Not clear)	Not clear (NR)<br><br>[NR]	Non-valvular	1100	**Warfarin**<br>1. INR 2-4.5 in <75 yrs.<br>2. INR 2.0-4.5 in >75 yrs.<br><br>**Antiplatelet (aspirin)**<br>3. 325mg (in <75 yrs.) od<br>4. 325mg (in >75 yrs.) od	37.2 (mean for age <75 years)<br><br>24 (mean for age >75 years)	NR	Efficacy-Stroke or systemic embolism, ischaemic stroke, MI, TIA<br><br>Safety-Intracranial bleeding, death (all causes)	27.6 (mean)
**WASPO(41)**<br><br>(Multicentre)<br><br>[UK]	Phase III<br><br>Not declared	>80 & <90 yrs. (Median 83 yrs.)<br><br>[47%]	Permanent non-valvular<br><br>ECG diagnosed	75	**Warfarin**<br>1. INR 2-3<br><br>**Antiplatelet (aspirin)**<br>2. 300mg od	12	69.2%	Efficacy-All stroke, TIA<br><br>Safety-Death (all causes)	12

AF = atrial fibrillation; NVAF = non-valvular atrial fibrillation; MI = myocardial infarction; TIA = transient ischaemic attack; PE = pulmonary embolism; INR = international normalized ratio; ECG = electrocardiogram; rand = randomised; od = once daily; bd = twice daily; Tmt = treatment; NR = not reported.

*Our results are based on 515 patients as reported in the results tables; the trial report is inconsistent in this regard.

References of the included trials

1. Connolly S, Pogue J, Hart R, et al. Clopidogrel plus aspirin versus oral anticoagulation for atrial fibrillation in the Atrial fibrillation Clopidogrel Trial with Irbesartan for prevention of Vascular Events (ACTIVE W): a randomised controlled trial. Lancet 2006;367(9526):1903-12.
2. Petersen P, Boysen G, Godtfredsen J, Andersen ED, Andersen B. Placebo-controlled, randomised trial of warfarin and aspirin for prevention of thromboembolic complications in chronic atrial fibrillation. The Copenhagen AFASAK study. Lancet 1989;1(8631):175-9.
3. Gullov AL, Koefoed BG, Petersen P, et al. Fixed minidose warfarin and aspirin alone and in combination vs adjusted-dose warfarin for stroke prevention in atrial fibrillation: Second Copenhagen Atrial Fibrillation, Aspirin, and Anticoagulation Study. Arch Intern Med 1998;158(14):1513-21.
4. Liu X, Huang H, Yu J, et al. Warfarin compared with aspirin for older Chinese patients with stable coronary heart diseases and atrial fibrillation complications. Int J Clin Pharmacol Ther 2014;52(6):454-9.
5. Boehringer Ingelheim. A dose response study of dabigatran etexilate (BIBR 1048) in pharmacodynamics and safety in patients with non-valvular atrial fibrillation in comparison to warfarin NCT01136408. 2014.
6. Chung N, Jeon HK, Lien LM, et al. Safety of edoxaban, an oral factor Xa inhibitor, in Asian patients with non-valvular atrial fibrillation. Thromb Haemost 2011;105(3):535-44.
7. Yamashita T, Koretsune Y, Yasaka M, et al. Randomized, multicenter, warfarin-controlled phase II study of edoxaban in Japanese patients with non-valvular atrial fibrillation. Circ J 2012;76(8):1840-7.
8. Weitz JI, Connolly SJ, Patel I, et al. Randomised, parallel-group, multicentre, multinational phase 2 study comparing edoxaban, an oral factor Xa inhibitor, with warfarin for stroke prevention in patients with atrial fibrillation. Thromb Haemost 2010;104(3):633-41.
9. Chen KP, Huang CX, Huang DJ, et al. Anticoagulation therapy in Chinese patients with non-valvular atrial fibrillation: a prospective, multi-center, randomized, controlled study. Chin Med J 2012;125(24):4355-60.
10. Lopes RD, Alexander JH, Al-Khatib SM, et al. Apixaban for reduction in stroke and other ThromboemboLic events in atrial fibrillation (ARISTOTLE) trial: design and rationale. Am Heart J 2010;159(3):331-9.
11. Granger CB, Alexander JH, McMurray JJ, et al. Apixaban versus warfarin in patients with atrial fibrillation. N Engl J Med 2011;365(11):981-92.
12. Easton JD, Lopes RD, Bahit MC, et al. Apixaban compared with warfarin in patients with atrial fibrillation and previous stroke or transient ischaemic attack: a subgroup analysis of the ARISTOTLE trial. Lancet Neurol 2012;11(6):503-11.
13. Hohnloser SH, Hijazi Z, Thomas L, et al. Efficacy of apixaban when compared with warfarin in relation to renal function in patients with atrial fibrillation: insights from the ARISTOTLE trial. Eur Heart J 2012;33(22):2821-30.
14. Al-Khatib SM, Thomas L, Wallentin L, et al. Outcomes of apixaban vs. warfarin by type and duration of atrial fibrillation: results from the ARISTOTLE trial. Eur Heart J 2013;34(31):2464-71.

15. McMurray JJ, Ezekowitz JA, Lewis BS, et al. Left ventricular systolic dysfunction, heart failure, and the risk of stroke and systemic embolism in patients with atrial fibrillation: insights from the ARISTOTLE trial. Circ Heart Fail 2013;6(3):451-60.
16. Flaker GC, Hohnloser S, Wojdyla D, et al. Apixaban is efficacious and safe in patients with at rial fibrillation using concomitant amiodarone: An analysis from the aristotle trial. J Am Coll Cardiol 2013;1):E317.
17. Garcia DA, Wallentin L, Lopes RD, et al. Apixaban versus warfarin in patients with atrial fibrillation according to prior warfarin use: results from the Apixaban for Reduction in Stroke and Other Thromboembolic Events in Atrial Fibrillation trial. Am Heart J 2013;166(3):549-58.
18. Bahit MC, Lopes RD, Wojdyla DM, et al. Apixaban in patients with atrial fibrillation and prior coronary artery disease: insights from the ARISTOTLE trial. Int J Cardiol 2013;170(2):215-20.
19. Alexander JH, Lopes RD, Thomas L, et al. Apixaban vs. warfarin with concomitant aspirin in patients with atrial fibrillation: insights from the ARISTOTLE trial. Eur Heart J 2014;35(4):224-32.
20. Hylek E, Held C, Alexander J, et al. Major bleeding in patients with atrial fibrillation receiving apixaban or warfarin: The ARISTOTLE trial (Apixaban for Reduction in Stroke and Other Thromboembolic Events in Atrial Fibrillation): Predictors, characteristics, and clinical outcomes. J Am Coll Cardiol 2014;63(20):2141-7.
21. Ogawa S, Shinohara Y, Kanmuri K. Safety and efficacy of the oral direct factor xa inhibitor apixaban in Japanese patients with non-valvular atrial fibrillation. -The ARISTOTLE-J study. Circ J 2011;75(8):1852-9.
22. Eikelboom JW, O'Donnell M, Yusuf S, et al. Rationale and design of AVERROES: apixaban versus acetylsalicylic acid to prevent stroke in atrial fibrillation patients who have failed or are unsuitable for vitamin K antagonist treatment. Am Heart J 2010;159(3):348-53.e1.
23. Hohnloser S, Yusuf S, Eikelboom J, et al. Apixaban in patients with atrial fibrillation and their risk for cardiovascular hospitalization: Insights from the AVERROES trial. Eur Heart J 2011;32:671.
24. Connolly SJ, Eikelboom J, Joyner C, et al. Apixaban in patients with atrial fibrillation. N Engl J Med 2011;364(9):806-17.
25. Diener HC, Eikelboom J, Connolly SJ, et al. Apixaban versus aspirin in patients with atrial fibrillation and previous stroke or transient ischaemic attack: a predefined subgroup analysis from AVERROES, a randomised trial. Lancet Neurol 2012;11(3):225-31.
26. Mant J, Hobbs FD, Fletcher K, et al. Warfarin versus aspirin for stroke prevention in an elderly community population with atrial fibrillation (the Birmingham Atrial Fibrillation Treatment of the Aged Study, BAFTA): a randomised controlled trial. Lancet 2007;370(9586):493-503.
27. Hu DY, Zhang HP, Sun YH, Jiang LQ. [The randomized study of efficiency and safety of antithrombotic therapy in nonvalvular atrial fibrillation: warfarin compared with aspirin]. Zhonghua Xin Xue Guan Bing Za Zhi 2006;34(4):295-8.
28. Ruff CT, Giugliano RP, Antman EM, et al. Evaluation of the novel factor Xa inhibitor edoxaban compared with warfarin in patients with atrial fibrillation: design and rationale for the Effective aNticoaGulation with factor xA next GEneration in Atrial Fibrillation-Thrombolysis In Myocardial Infarction study 48 (ENGAGE AF-TIMI 48). Am Heart J 2010;160(4):635-41.
29. Giugliano RP, Ruff CT, Braunwald E, et al. Edoxaban versus warfarin in patients with atrial fibrillation. N Engl J Med 2013;369(22):2093-104.

30. Connolly SJ, Eikelboom J, Dorian P, et al. Betrixaban compared with warfarin in patients with atrial fibrillation: results of a phase 2, randomized, dose-ranging study (Explore-Xa). Eur Heart J 2013;34(20):1498-505.
31. Hori M, Matsumoto M, Tanahashi N, et al. Rivaroxaban vs. warfarin in Japanese patients with atrial fibrillation - the J-ROCKET AF study. Circ J 2012;76(9):2104-11.
32. Hellemons BS, Langenberg M, Lodder J, et al. Primary prevention of arterial thromboembolism in non-rheumatic atrial fibrillation in primary care: randomised controlled trial comparing two intensities of coumarin with aspirin. BMJ 1999;319(7215):958-64.
33. Ezekowitz MD, Reilly PA, Nehmiz G, et al. Dabigatran with or without concomitant aspirin compared with warfarin alone in patients with nonvalvular atrial fibrillation (PETRO Study). Am J Cardiol 2007;100(9):1419-26.
34. Connolly SJ, Ezekowitz MD, Yusuf S, et al. Dabigatran versus warfarin in patients with atrial fibrillation. N Engl J Med 2009;361(12):1139-51.
35. Connolly SJ, Ezekowitz MD, Yusuf S, Reilly PA, Wallentin L. Newly identified events in the RE-LY trial. N Engl J Med 2010;363(19):1875-6.
36. Rivaroxaban-Once daily, oral, direct factor Xa inhibition Compared with vitamin K antagonism for prevention of stroke and Embolism Trial in Atrial Fibrillation: Rationale and Design of the ROCKET AF study. Am Heart J 2010;159(3):340-7 e1.
37. Patel MR, Mahaffey KW, Garg J, et al. Rivaroxaban versus warfarin in nonvalvular atrial fibrillation. N Engl J Med 2011;365(10):883-91.
38. Hankey GJ, Patel MR, Stevens SR, et al. Rivaroxaban compared with warfarin in patients with atrial fibrillation and previous stroke or transient ischaemic attack: a subgroup analysis of ROCKET AF. Lancet Neurol 2012;11(4):315-22.
39. Mahaffey KW, Wojdyla D, Hankey GJ, et al. Clinical outcomes with rivaroxaban in patients transitioned from vitamin K antagonist therapy: a subgroup analysis of a randomized trial.[Summary for patients in Ann Intern Med. 2013 Jun 18;158(12):I-28; PMID: 23778921]. Ann Intern Med 2013;158(12):861-8.
40. Stroke Prevention in Atrial Fibrillation Investigators. Warfarin versus aspirin for prevention of thromboembolism in atrial fibrillation: Stroke Prevention in Atrial Fibrillation II Study. Lancet 1994;343(8899):687-91.
41. Rash A, Downes T, Portner R, Yeo WW, Morgan N, Channer KS. A randomised controlled trial of warfarin versus aspirin for stroke prevention in octogenarians with atrial fibrillation (WASPO). Age Ageing 2007;36(2):151-6.
