# Supplementary material for: Oral anticoagulants for prevention of stroke in atrial fibrillation: systematic review, network meta-analysis, and cost effectiveness analysis
Source: BMJ. 2017 Nov 28;359:j5058. doi: 10.1136/bmj.j5058 (PMC5704695; doi:10.1136/bmj.j5058)
Supplement: Supplementary file 4 — Appendix: Supplementary materials [file lopj038668.ww4.pdf]

Appendix 4: Additional outcomes reported by 23 included randomised trials in stroke prevention in AF

Study	Study size	Reporting pattern	TIA	Minor ischemic stroke	Major ischemic stroke	Fatal stroke	PE	Hospital admission	Fatal bleeding	EC minor bleeding	Arterial event	CR non-major bleeding	Cardio-vascular deaths
ACTIVE W	6706	1							18				
AF-ASA-VKA-CHINA	101	2							2				
AF-DABIG-VKA-JAPAN	166	2											
AF-EDOX-VKA-ASIA	234	2										9	
AF-EDOX-VKA-JAPAN	519	2										15	
AF-EDOX-VKA-MULTI	1143	2						12				49	8
AF-VKA-ASA-CHINA	440	2	13										
AFASAK	671	2	2	1		4							
AFASAK II	339	1	3			2							
ARISTOTLE	18140	3											
ARISTOTLE-J	218	2	1									5	
AVERROES	5599	1							10			180	180
BAFTA	973	1											
Chinese ATAFS	704	2											
ENGAGE AF-TIMI 48	21026	2				239			112			3579	1668
EXPLORE-Xa	508	2										12	
J-ROCKET AF	1278	2											8
PATAF	272	1		2	5						8		18
PETRO	515	2											
RE-LY	18113	2					43	7199		956			880
ROCKET AF	14236	2							82			2336	
SPAF II	1100	3	25										
WASPO	75	2	1										
Total	93076		45	3	5	245	43	7211	224	956	8	6185	2762

TIA: transient ischaemic attack; PE: pulmonary embolism; EC: extracranial; CR: clinically relevant.

Reporting patterns: (1) Number of patients whose first event is of a given type, patients censored thereafter; (2) Number of patients experiencing at least one event of each given type; (3) Total number of events of each type.
