# Supplementary material for: Oral anticoagulants for prevention of stroke in atrial fibrillation: systematic review, network meta-analysis, and cost effectiveness analysis
Source: BMJ. 2017 Nov 28;359:j5058. doi: 10.1136/bmj.j5058 (PMC5704695; doi:10.1136/bmj.j5058)
Supplement: Supplementary file 5 — Appendix: Supplementary materials [file lopj038668.ww5.pdf]

Appendix 5: Risk of bias assessments for each outcome

A5.0: List of distinct interventions examined by included randomised trials in stroke prevention in AF

1	Warfarin (INR 2-3)
2	Warfarin (INR 1.6-3)
3	Warfarin (INR 3-4 od)
4	Antiplatelet (<150mg od)
5	Antiplatelet (≥150mg od)
6	Dabigatran (50mg bd) + Aspirin (81mg bd)
7	Dabigatran (50mg bd) + Aspirin (325mg bd)
8	Dabigatran (150mg bd) + Aspirin (81mg bd)
9	Dabigatran (150mg bd) + Aspirin (325mg bd)
10	Dabigatran (300mg bd) + Aspirin (81mg bd)
11	Dabigatran (300mg bd) + Aspirin (325mg bd)
12	Apixaban (2.5mg bd)
13	Apixaban (5mg bd)
14	Dabigatran (50mg bd)
15	Dabigatran (110mg bd)
16	Dabigatran (150mg bd)
17	Dabigatran (300mg bd)
18	Betrixaban (40mg od)
19	Betrixaban (60mg od)
20	Betrixaban (80mg od)
21	Edoxaban (30mg od)
22	Edoxaban (45mg od)
23	Edoxaban (60mg od)
24	Edoxaban (30mg bd)
25	Edoxaban (60mg bd)
26	Rivaroxaban (15mg od)
27	Rivaroxaban (20mg od)

A5.1: Included trials and risk of bias assessment for stroke or systemic embolism

Study	Interventions compared	Sequence generation	Allocation concealment	Blinding of participants and personnel	Blinding of outcome assessment	Incomplete outcome data	Selective reporting
ACTIVE W	1, 4	+	+	–	+	+	?
AFASAK	1, 4	+	+	–	?	+	?
AFASAK II	1, 5	+	?	–	+	+	?
AF-ASA-VKA-CHINA	2, 4	?	–	–	–	+	?
AF-DABIG-VKA-JAPAN	2, 15, 16	?	?	?	?	?	?
AF-EDOX-VKA-ASIA	1, 21, 23	+	+	–	+	+	+
AF-EDOX-VKA-JAPAN	2, 21, 22, 23	+	?	–	?	+	+
AF-EDOX-VKA-MULTI	1, 21, 23, 24, 25	+	+	–	?	+	+
AF-VKA-ASA-CHINA	1, 5	+	?	?	?	?	?
ARISTOTLE	1, 13	+	?	+	+	+	+
ARISTOTLE-J	1, 12, 13	?	?	–	+	+	+
AVERROES	4, 13	+	+	+	+	+	+
BAFTA	1, 4	+	+	–	+	+	+
Chinese ATAFS	2, 5	?	?	?	?	+	?
ENGAGE AF-TIMI 48	1, 21, 23	+	+	+	+	+	+
EXPLORE-Xa	1, 18, 19, 20	?	?	–	+	+	+
J-ROCKET AF	2, 26	+	+	+	?	+	+
PATAF	1, 5	+	+	?	+	?	+
PETRO	1, 6, 7, 8, 9 10, 11, 14, 16, 17	?	?	–	?	+	+
RE-LY	1, 15, 16	+	+	–	+	+	+
ROCKET AF	1, 27	+	+	+	?	+	+
SPAF II	1, 5	+	?	–	+	+	?
WASPO	1, 5	+	+	–	–	+	?

A5.2: Included trials and risk of bias assessment for ischaemic stroke

Study	Interventions compared	Sequence generation	Allocation concealment	Blinding of participants and personnel	Blinding of outcome assessment	Incomplete outcome data	Selective reporting
ACTIVE W	1, 4	+	+	–	+	+	?
AFASAK II	1, 5	+	?	–	+	+	?
AF-ASA-VKA-CHINA	2, 4	?	–	–	–	+	?
AF-VKA-ASA-CHINA	1, 5	+	?	?	?	?	?
ARISTOTLE	1, 13	+	?	+	+	+	+
ARISTOTLE-J	1, 12, 13	?	?	–	+	+	+
AVERROES	4, 13	+	+	+	+	+	+
ENGAGE AF-TIMI 48	1, 21, 23	+	+	+	+	+	+
EXPLORE-Xa	1, 18, 19, 20	?	?	–	+	+	+
J-ROCKET AF	2, 26	+	+	+	?	+	+
PATAF	1, 5	+	+	?	+	?	+
RE-LY	1, 15, 16	+	+	–	+	+	+
ROCKET AF	1, 27	+	+	+	?	+	+
SPAF II	1, 5	+	?	–	+	+	?

A5.3: Included trials and risk of bias assessment for myocardial infarction

Study	Interventions compared	Sequence generation	Allocation concealment	Blinding of participants and personnel	Blinding of outcome assessment	Incomplete outcome data	Selective reporting
ACTIVE W	1, 4	+	+	–	+	+	?
AFASAK II	1, 5	+	?	–	+	+	?
AF-ASA-VKA-CHINA	2, 4	?	–	–	–	+	?
AF-EDOX-VKA-MULTI	1, 21, 23, 24, 25	+	+	–	?	+	+
ARISTOTLE	1, 13	+	?	+	+	+	+
ARISTOTLE-J	1, 12, 13	?	?	–	+	+	+
AVERROES	4, 13	+	+	+	+	+	+
BAFTA	1, 4	+	+	–	+	+	+
ENGAGE AF-TIMI 48	1, 21, 23	+	+	+	+	+	+
EXPLORE-Xa	1, 18, 19, 20	?	?	–	+	+	+
J-ROCKET AF	2, 26	+	+	+	?	+	+
PATAF	1, 5	+	+	?	+	?	+
RE-LY	1, 15, 16	+	+	–	+	+	+
ROCKET AF	1, 27	+	+	+	?	+	+
SPAF II	1, 5	+	?	–	–	+	?

A5.4: Included trials and risk of bias assessment for all-cause mortality

Study	Interventions compared	Sequence generation	Allocation concealment	Blinding of participants and personnel	Blinding of outcome assessment	Incomplete outcome data	Selective reporting
ACTIVE W	1, 4	+	+	–	+	+	?
AFASAK	1, 4	+	+	–	?	+	?
AFASAK II	1, 5	+	?	–	+	+	?
AF-ASA-VKA-CHINA	2, 4	?	–	–	–	+	?
AF-VKA-ASA-CHINA	1, 5	+	?	–	?	?	?
ARISTOTLE	1, 13	+	?	+	+	+	+
ARISTOTLE-J	1, 12, 13	?	?	–	+	+	+
AVERROES	4, 13	+	+	+	+	+	+
BAFTA	1, 4	+	+	–	+	+	+
Chinese ATAFS	2, 5	?	?	?	?	+	?
ENGAGE AF-TIMI 48	1, 21, 23	+	+	+	+	+	+
EXPLORE-Xa	1, 18, 19, 20	?	?	–	+	+	+
J-ROCKET AF	2, 26	+	+	+	?	+	+
PATAF	1, 5	+	+	?	+	?	+
RE-LY	1, 15, 16	+	+	–	+	+	+
ROCKET AF	1, 27	+	+	+	+	+	+
SPAF II	1, 5	+	?	–	–	+	?
WASPO	1, 5	+	+	–	–	+	?

A5.5: Included trials and risk of bias assessment for major bleeding

Study	Interventions compared	Sequence generation	Allocation concealment	Blinding of participants and personnel	Blinding of outcome assessment	Incomplete outcome data	Selective reporting
ACTIVE W	1, 4	+	+	–	+	+	?
AFASAK II	1, 5	+	?	–	+	+	?
AF-ASA-VKA-CHINA	2, 4	?	–	–	–	+	?
AF-DABIG-VKA-JAPAN	2, 15, 16	?	?	?	?	?	?
AF-EDOX-VKA-ASIA	1, 21, 23	+	+	–	+	+	+
AF-EDOX-VKA-JAPAN	2, 21, 22, 23	+	?	–	?	+	+
AF-EDOX-VKA-MULTI	1, 21, 23, 24, 25	+	+	–	+	+	+
AF-VKA-ASA-CHINA	1, 5	+	?	–	?	?	?
ARISTOTLE	1, 13	+	?	+	+	?	+
ARISTOTLE-J	1, 12, 13	?	?	–	+	+	+
AVERROES	4, 13	+	+	+	+	+	+
BAFTA	1, 4	+	+	–	+	+	+
ENGAGE AF-TIMI 48	1, 21, 23	+	+	+	+	+	+
EXPLORE-Xa	1, 18, 19, 20	?	?	–	+	+	+
PETRO	1, 6, 7, 8, 9 10, 11, 14, 16, 17	?	?	–	+	+	+
RE-LY	1, 15, 16	+	+	–	+	+	+
ROCKET AF	1, 27	+	+	+	?	+	+
WASPO	1, 5	+	+	–	–	+	?

A5.6: Included trials and risk of bias assessment for intracranial bleeding

Study	Interventions compared	Sequence generation	Allocation concealment	Blinding of participants and personnel	Blinding of outcome assessment	Incomplete outcome data	Selective reporting
AFASAK II	1, 5	+	?	−	+	+	?
ARISTOTLE	1, 13	+	?	+	+	?	+
AVERROES	4, 13	+	+	+	+	+	+
ENGAGE AF-TIMI 48	1, 21, 23	+	+	+	+	+	+
RE-LY	1, 15, 16	+	+	−	+	+	+
J-ROCKET AF	2, 26	+	+	+	?	+	+
ROCKET AF	1, 27	+	+	+	?	+	+
SPAF II	1, 5	+	?	−	−	+	?

A5.7: Included trials and risk of bias assessment for clinically relevant bleeding

Study	Interventions compared	Sequence generation	Allocation concealment	Blinding of participants and personnel	Blinding of outcome assessment	Incomplete outcome data	Selective reporting
AF-DABIG-VKA-JAPAN	2, 15, 16	?	?	?	?	?	?
AF-EDOX-VKA-ASIA	1, 21, 23	+	+	–	+	+	+
AF-EDOX-VKA-JAPAN	2, 21, 22, 23	+	?	–	?	+	+
AF-EDOX-VKA-MULTI	1, 21, 23, 24, 25	+	+	–	+	+	+
ARISTOTLE	1, 13	+	?	+	+	?	+
ARISTOTLE-J	1, 12, 13	?	?	–	+	+	+
AVERROES	4, 13	+	+	+	+	+	+
ENGAGE AF-TIMI 48	1, 21, 23	+	+	+	+	+	+
EXPLORE-Xa	1, 18, 19, 20	?	?	–	+	+	+
J-ROCKET AF	2, 26	+	+	+	?	+	+
PETRO	1, 6, 7, 8, 9 10, 11, 14, 16, 17	?	?	–	+	+	+
ROCKET AF	1, 27	+	+	+	?	+	+
