# Supplementary material for: Oral anticoagulants for prevention of stroke in atrial fibrillation: systematic review, network meta-analysis, and cost effectiveness analysis
Source: BMJ. 2017 Nov 28;359:j5058. doi: 10.1136/bmj.j5058 (PMC5704695; doi:10.1136/bmj.j5058)
Supplement: Supplementary file 6 — Appendix: Supplementary materials [file lopj038668.ww6.pdf]

Appendix 6: Effect modifiers examined in stroke prevention in AF

Study	Intervention	Mean time in therapeutic range (INR)	Mean age (years)	Percentage of males	Mean CHADS2 score
ACTIVE W	Antiplatelet (Clopidogrel 75mg + (aspirin 75-100mg) od	NA	70.2	67	2
ACTIVE W	Warfarin (INR 2-3, some patients may have received other vitamin K antagonists)	63.8%	70.2	66	2
AFASAK	Warfarin (INR 2-3)	73%	72.8	53	NR
AFASAK	Aspirin 75mg od	NA	75.1	55	NR
AFASAK II	Aspirin 300mg od	NA	73.1	65	NR
AFASAK II	Warfarin (INR 2-3)	73%	73.2	57	NR
AF-ASA-VKA-CHINA	Warfarin (INR 1.6-2.5)	NR	60.8	2.9	NR
AF-ASA-VKA-CHINA	Aspirin 100mg od	NA	60	2.8	NR
AF-DABIG-VKA-JAPAN	Dabigatran110mg bd	NA	NR	NR	NR
AF-DABIG-VKA-JAPAN	Dabigatran 150mg bd	NA	NR	NR	NR
AF-DABIG-VKA-JAPAN	Warfarin (INR 2-3, ≥1.6 to ≤2.6 in ≥70 yrs)	NR	NR	NR	NR
AF-EDOX-VKA-ASIA	Edoxaban 30mg od	NA	64.9	64.6	2
AF-EDOX-VKA-ASIA	Edoxaban 60mg od	NA	65.9	68.8	1.9
AF-EDOX-VKA-ASIA	Warfarin (INR 2-3)	45.1%	64.5	62.7	1.8
AF-EDOX-VKA-JAPAN	Edoxaban 30mg od	NA	69.4	84	1.9
AF-EDOX-VKA-JAPAN	Edoxaban 45mg od	NA	69.5	81.3	2.1
AF-EDOX-VKA-JAPAN	Edoxaban 60mg od	NA	68.4	81.7	2.1
AF-EDOX-VKA-JAPAN	Warfarin (INR 2-3, 1.6-2.6 in ≥70 yrs.)	83% (≥70 yrs.) 73% (<70 yrs.)	68.8	82.9	2.2
AF-EDOX-VKA-MULTI	Edoxaban 30mg od	NA	65.2	59.6	3.12
AF-EDOX-VKA-MULTI	Edoxaban 30mg bd	NA	64.8	61.5	3.1
AF-EDOX-VKA-MULTI	Edoxaban 60mg od	NA	64.9	66.2	3.06
AF-EDOX-VKA-MULTI	Edoxaban 60mg bd	NA	64.7	63.3	3.06
AF-EDOX-VKA-MULTI	Warfarin (INR 2-3)	49.7%	66	60.4	3.03
AF-VKA-ASA-CHINA	Warfarin (INR 2.1-2.5)	NR	66.8	63.2	NR
AF-VKA-ASA-CHINA	Aspirin 200mg od	NA	67.6	59.2	NR

Study	Intervention	Mean time in therapeutic range (INR)	Mean age (years)	Percentage of males	Mean CHADS2 score
ARISTOTLE	Apixaban 5mg bd	NA	70	64.5	2.1
ARISTOTLE	Warfarin (INR 2-3)	62.2%	70	65	2.1
ARISTOTLE-J	Apixaban 2.5mg bd	NA	69.3	85.1	1.8
ARISTOTLE-J	Apixaban 5mg bd	NA	70	82.4	2.1
ARISTOTLE-J	Warfarin (INR 2-3, 2-2.6 in ≥70 yrs.)	60%	71.7	81.1	1.9
AVERROES	Apixaban 5mg bd	NA	70	59	2
AVERROES	Aspirin 81-324mg od	NA	70	58	2.1
BAFTA	Aspirin 75mg od	NA	81.5	54	2.33
BAFTA	Warfarin (INR 2-3)	67%	81.5	55	2.34
Chinese ATAFS	Warfarin (INR 2-3, 1.6-2.5 in >75 yrs.)	NR	NR	NR	NR
Chinese ATAFS	Aspirin 150-160mg od	NA	NR	NR	NR
ENGAGE AF-TIMI 48	Edoxaban 30mg od	NA	72	62.5	2.8
ENGAGE AF-TIMI 48	Edoxaban 60mg od	NA	72	62.1	2.8
ENGAGE AF-TIMI 48	Warfarin (INR 2-3)	64.9%	72	61.2	2.8
EXPLORE-Xa	Betrixaban 40mg od	NA	73.3	62.2	2.59
EXPLORE-Xa	Betrixaban 60mg od	NA	73.8	63.8	2.48
EXPLORE-Xa	Betrixaban 80mg od	NA	72	70.1	2.06
EXPLORE-Xa	Warfarin (INR 2-3)	63.4%	72.7	70.1	2.51
J-ROCKET AF	Rivaroxaban 15mg od	NA	71	82.9	3.27
J-ROCKET AF	Warfarin (INR 2-3, 1.6-2.6 in ≥70 yrs.)	65%	71.2	78.2	3.22
PATAF	Aspirin150mg od	NA	70.8	48	NR
PATAF	Dicoumarol (INR 2.5-3.5, some patients received other coumarins – phenprocoumon or acenocoumarol)	NR	70	44	NR
PETRO	Dabigatran 50mg bd	NA	70	80	NR
PETRO	Dabigatran 50mg + Aspirin 81mg bd	NA	NR	NR	NR
PETRO	Dabigatran 50mg + Aspirin 325mg bd	NA	NR	NR	NR
PETRO	Dabigatran 150mg bd	NA	70	81.3	NR
PETRO	Dabigatran 150mg + Aspirin 81mg bd	NA	NR	NR	NR

Study	Intervention	Mean time in therapeutic range (INR)	Mean age (years)	Percentage of males	Mean CHADS2 score
PETRO	Dabigatran 150mg + Aspirin 325mg bd	NA	NR	NR	NR
PETRO	Dabigatran 300mg bd	NA	69.5	82.6	NR
PETRO	Dabigatran 300mg + Aspirin 81mg bd	NA	NR	NR	NR
PETRO	Dabigatran 300mg + Aspirin 325mg bd	NA	NR	NR	NR
PETRO	Warfarin (INR 2-3)	57.2%	69	84.3	NR
RE-LY	Dabigatran 110mg bd	NA	71.4	64.3	2.1
RE-LY	Dabigatran 150mg bd	NA	71.5	63.2	2.2
RE-LY	Warfarin (INR 2-3)	64%	71.6	63.3	2.1
ROCKET AF	Rivaroxaban 20mg od	NA	73	60.3	3.48
ROCKET AF	Warfarin (INR 2-3)	55%	73	60.3	3.46
SPAF II	Warfarin (INR 2-4.5 in <75yrs.)	NR	65	75	NR
SPAF II	Aspirin 325mg (in <75 yrs.)	NA	64	76	NR
SPAF II	Warfarin (INR 2-4.5 in ≥75yrs.)	NR	80	59	NR
SPAF II	Aspirin 325mg (in ≥75 yrs.)	NA	80	58	NR
WASPO	Warfarin (INR 2-3)	69.2%	83.5	39	NR
WASPO	Aspirin 300mg od	NA	82.6	54	NR

AF = atrial fibrillation; INR = international normalized ratio; NA; not applicable; NR = not reported, od = once daily; bd = twice daily, asp = aspirin
