# Supplementary figures and images for: Oral anticoagulants for prevention of stroke in atrial fibrillation: systematic review, network meta-analysis, and cost effectiveness analysis
Source: BMJ. 2017 Nov 28;359:j5058. doi: 10.1136/bmj.j5058 (PMC5704695; doi:10.1136/bmj.j5058)

Appendix 7: Incremental cost-effectiveness plane, relative to warfarin (INR 2-3) as reference

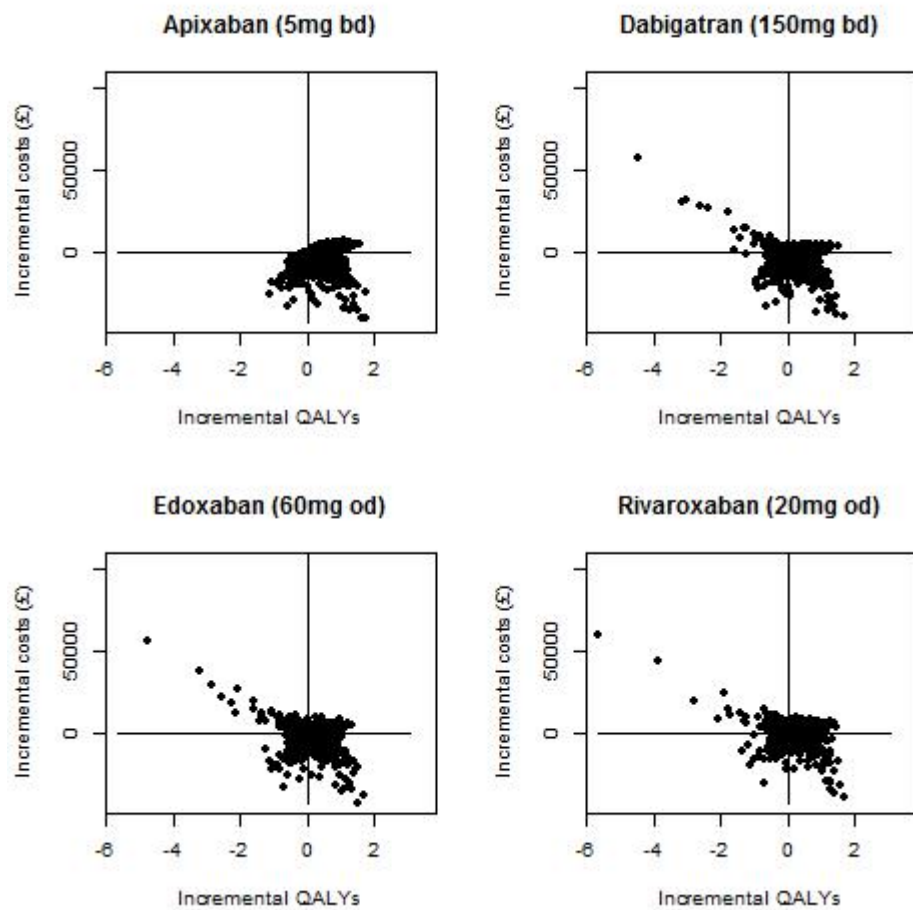

Supplement: Supplementary file 7 — Appendix: Supplementary materials [file lopj038668.ww7.pdf]
